# Assessment of the potential role of atmospheric particulate pollution and airborne transmission in intensifying the first wave pandemic impact of SARS-CoV-2/COVID-19 in Northern Italy

**DOI:** 10.1007/s42865-020-00024-3

**Published:** 2020-12-21

**Authors:** Paolo Di Girolamo

**Affiliations:** Scuola di Ingegneria, Università degli Studi della Basilicata, Viale dell’Ateneo Lucano, 10, 85100 Potenza, Italy

**Keywords:** COVID-19, SARS-CoV-2, Environmental pollution, PM_2.5_, PM_10_, Epidemiologic parameters, Airborne virus transmission

## Abstract

The severe acute respiratory syndrome coronavirus 2 (SARS-CoV-2), which exploded in Wuhan (Hebei Region, China) in late 2019, has later spread around the world, causing pandemic effects on humans. During the first wave of the pandemic, Italy, and especially its Northern regions around the Po Valley, faced severe consequences in terms of infected individuals and casualties (more than 31,000 deaths and 255,000 infected people by mid-May 2020). While the spread and effective impact of the virus is primarily related to the lifestyles and social habits of the different human communities, environmental and meteorological factors also play a role. Among these, particulate pollution may directly impact the human respiratory system or act as virus carrier, thus behaving as potential amplifying factor in the pandemic spread of SARS-CoV-2. Enhanced levels of PM_2.5_ and PM_10_ particles in Northern Italy were observed over the 2-month period preceding the virus pandemic spread. Threshold levels for PM_10_ (< 50 μg/m^3^) were exceeded on 20–35 days over the period January–February 2020 in many areas in the Po Valley, where major effects in terms of infections and casualties occurred, with levels in excess of 80 μg/m^3^ occasionally observed in the 1–3 weeks preceding the contagious activation around February 25, 2020. Threshold values for PM_2.5_ indicated in WHO air quality guidelines (< 25 μg/m^3^) were exceeded on more than 40 days over the period January–February 2020 in large portions of the Po Valley, with levels up to 70 μg/m^3^ observed in the weeks preceding the contagious activation. In this paper, PM_10_ particle measurements are compared with epidemiologic parameters’ data. Specifically, a statistical analysis is carried out to correlate the infection rate, or incidence of the pathology, the mortality rate, and the case fatality rate with PM concentrations. The study considers epidemiologic data for all 110 Italian provinces, as reported by the Italian Statistics Institute, over the period 20 February–31 March 2020. Corresponding PM_10_ concentrations covering the period 15–26 February 2020 were collected from the network of air quality monitoring stations run by different regional and provincial environment agencies. The case fatality rate is found to be highly correlated to the average PM_10_ concentration, with a correlation coefficient of 0.89 and a slope of the regression line of (6.7 ± 0.3) × 10^−3^ m^3^/μg, which implies a doubling (from 3 to 6%) of the mortality rate of infected patients for an average PM_10_ concentration increase from 22 to 27 μg/m^3^. Infection and mortality rates are also found to be correlated with PM_10_ concentrations, with correlation coefficients being 0.82 and 0.80, respectively, and the slopes of the regression lines indicating a doubling (from 1 to 2‰) of the infection rate and a tripling (from 0.1 to 0.3‰) of the mortality rate for an average PM_10_ concentration increase from 25 to 29 μg/m^3^. Considerations on the exhaled particles’ sizes, their concentrations and residence times, the transported viral dose and the minimum infective dose, in combination with PM_2.5_ and PM_10_ pollution measurements and an analytical microphysical model, allowed assessing the potential role of airborne transmission through virus-laden PM particles, in addition to droplet and the traditional airborne transmission, in conveying SARS-CoV-2 in the human respiratory system. In specific circumstances which can be found in indoor environments, the number of small potentially infectious particles coalescing on PM_2.5_ and PM_10_ particles is estimated to exceed the number of infectious particles needed to activate COVID-19 infection in humans.

## Introduction

PM_2.5_ and PM_10_ are particles with an aerodynamic diameter smaller than 2.5 and 10 μm, respectively, which are often present in the air. These small particles can be either organic or inorganic and can be present in both solid and liquid phase. They are capable of adsorbing on their surface various substances with toxic properties such as sulfates, nitrates, metals, and volatile compounds (Park et al. 2018).

Suspended PM_2.5_ and PM_10_ particles have a significant impact on human health, the higher being their concentration, the greater being their health impact (Dockery et al. 1993; Pope et al. 1995; Brunekreef 1997; Hoek et al. 2002). More specifically, atmospheric aerosols play an important role in triggering pro-inflammation and oxidation mechanisms of the lungs. Prolonged exposure to PM_2.5_ and PM_10_ particles has been found to be linked to acute respiratory inflammation and immunological alterations (Li et al. 2018; Losacco and Perillo 2018). Aerosols can represent an important vehicle for virus transmission (Sattar and Ijaz 1987; Fabian et al. 2008; Tellier 2009).

In the present paper, we report a statistical analysis correlating SARS-CoV-2/COVID-19 epidemiologic parameters to PM_10_ particle concentration measurements. This is not the first attempt to correlate COVID-19 epidemiologic parameters with particulate matter pollution. In a recent paper by Setti et al. (2020a), the relationship between the PM_10_ daily limit value exceedances and COVID-19 infection rate was investigated for the different Italian provinces and the statistical significance of results was assessed based on an estimate of the *p* value. The number of exceedances of PM_10_ daily limit value is certainly a quantitative indicator of the frequency of high pollution events, but it is not an effective quantitative indicator of the time persistency of pollution conditions. Additionally, Setti et al. (2020a) focused only on one epidemiologic parameter, i.e., the infection rate, but did not consider other important parameters as the mortality rate and the case fatality rate, which are of paramount importance for an effective assessment of a pandemic outbreak impact. The study by Setti et al. (2020a) considers the infection rate over a quite short time interval (24 February–13 March 2020); this interval represents a brief sub-portion of the lockdown period imposed to Italy. Furthermore, this time interval is a small sub-portion of the period characterized by the highest number of infections and deaths, thus resulting to be not particularly effective in representing the highest pandemic outbreak period of the pandemic.

In a recent paper by Borro et al. (2020), the variability of the infection rate, the mortality rate, and the case fatality rate as a function of particle concentration was estimated for PM_2.5_ particles only, recognizing a primary role of these smaller particles in inducing an over-expression of the angiotensin conversion enzyme 2 (ACE-2) in the human respiratory system (see papers by Gemmati et al. 2020; Devaux et al. 2020; Bunyavanich et al. 2020; Leung et al. 2020), and consequently in enhancing COVID-19 epidemiologic impact. In the present paper, we extend the analysis to PM_10_ particles.

Furthermore, in the study by Borro et al. (2020), particulate matter measurements from a single station for each province were considered, while in the present study, we consider measurements from all ground stations present within each province territory, which allows to account for the natural variability of the particulate loading within the single province territories, including urban, semi-urban, and rural areas. In fact, particulate concentration variability within each province territory may be large; this variability may severely affect the degree of correlation between epidemiologic parameters and atmospheric pollution and needs to be properly accounted for. For this purpose, particulate concentration variability, i.e., its standard deviation, within each province territory is used as a weighting factor in the present statistical analysis correlating epidemiologic factors with PM_10_ concentrations.

The paper outline is the following. Section 2 shortly describes compositional, size and microphysical properties of PM_2.5_ and PM_10_ particles. Section 3 illustrates the possible interaction mechanisms of aerosol particles with the human respiratory system. Section 4 provides an assessment of the potential role of airborne transmission through virus-laden PM particles. Section 5 illustrates the different datasets and sensors used in the study. Section 6 illustrates the evolution of PM_2.5_ and PM_10_ concentrations shortly before the pandemic outbreak and results in terms of correlations between epidemiologic parameters and PM concentrations. Finally, Section 7 provides a summary of all results and some perspectives for a possible future continuation of this research effort.

## PM_2.5_ and PM_10_ particles: their origin, composition, size and microphysical properties, and residence times

Particulate matter (PM) is the term typically used to refer to solid and liquid particles present in the air. Some of these particles are formed naturally, originating from volcanoes, forest and grassland fires, living vegetation, soil dust storms, and sea spray. However, most atmospheric PM_2.5_ and PM_10_ sources in polluted environments are linked to human activities. Generally, PM_2.5_ particles are formed from high temperature processes, such as vehicular exhaust, oil and coal combustion processes (internal combustion engines, heating systems, industrial activities, incinerators and thermoelectric power plants, biomass burning), industrial processes, and atmospheric chemical reactions (Harrison et al. 2003; Samara et al. 2003). PM_10_ particles are usually produced through attrition processes, including mechanical abrasion of crustal material and re-suspension of road and soil dust and brake and tire wear from vehicles (Allen et al. 2001). PM_10_ contains PM_2.5_, and—as such—contains not only coarse particles from natural sources and those generated through attrition and abrasion processes but also the smaller particles generated by combustion.

For example, a recent study on air pollution in the Lombardy region, one of the most industrialized areas in Northern Italy where the maximum permitted PM_10_ concentration threshold is frequently exceeded, revealed that the primary sources of PM_10_ particles are wood biomass combustion (pellet or wood stoves), responsible for 45% of the particles present in the air, diesel engines, contributing with 14%, while 13% results from particles detaching from brake pads and tires (ARPA Lombardia 2017). Another important source of PM_2.5_ and PM_10_ particles in this region is represented by the degradation of road surface asphalt. Obviously, the above numbers are considered to be mean values, with the geographical distribution of sources being characterized by a large variability within the region territory. Furthermore, natural sources of particle matter are important in this region, especially those associated with Saharan dust outbreaks, wildfires, and pollen.

Secondary aerosol formation, together with long-range atmospheric transport, represents an additional important source of PM_2.5_ particles. Secondary components contribute to PM_2.5_ and PM_10_ particle formation through chemical reactions, coagulation, and other mechanisms. For example, secondary PM_2.5_ and PM_10_ particles can be formed form ammonia (NH_3_) and inorganic acid gases, in combination with NOx and VOC compounds (Marais et al. 2016; Guerra et al. 2014; and Kim et al. 2018).

The definition of PM_2.5_ and PM_10_ particles based on their aerodynamic diameter provides an upper size limit for their dimensions, but does not provide any specific information on particles’ size distribution. PM_2.5_ and PM_10_ measurements are typically carried out with filtration samplers equipped with size-selective inlets capable to discriminate particles smaller than 2.5 and 10 μm, respectively. Particle number, volume, and mass concentration vary as a function of radius and a comprehensive evaluation of PM potential impact on human health requires an accurate assessment of their size distributions. While information on particle size distribution cannot be inferred from filter-based samples, several literature papers indicate that PM mass distribution in urban background conditions has a predominance of fine particle (PM_2.5_) mass. In industrial areas, the fine mode dominates in combustion-related activities, while the coarse fraction (PM_10–2.5_) is dominant in the case of mechanical activities (Taiwo et al. 2014; Pandolfi et al. 2011), with particle size distributions often found to be bimodal (Seinfeld and Pandis 1998; Verrilli et al. 2010).

Potential effects of suspended PM particles on human health are strongly dependent on their atmospheric life time, or residence time. Atmospheric residence time of PM particles is primarily dependent on their sizes and on atmospheric conditions. Vecchi et al. (2007) reported a typical residence time for atmospheric PM_10_ particles in urban areas of 10–12 h, while finer aerosols’ residence time was estimated to be 18–38 h (Vecchi et al. 2005). However, actual values may be larger especially for finer aerosols for which the residence time depends on the variability of precipitation events (Jaenicke 1982).

## Considerations on inhaled aerosol particles and their potential interaction with the human respiratory system

### General remarks

Suspended aerosols have a significant impact on human health. A mortality analysis carried out by Cui et al. (2003) for the previous coronavirus SARS (SARS-CoV-1) in China pointed out that patients in regions with moderated air pollution levels were more likely to die than those living in regions with low air pollution levels.

Aerosols represent an important vehicle for virus transmission (Sattar and Ijaz 1987; Fabian et al. 2008; Tellier 2009). In order for virus transmission through aerosols to occur, PM particles must carry a sufficient amount of the infectious virus and the virus must survive and remain infectious in the carrier particle for a sufficiently long time before it reaches a susceptible host cell and initiate infection. In general, airborne infectious viruses are difficult to monitor due to their extremely low concentrations in air and because of the inadequacy and lack of accuracy of currently used air samplers.

In general, the primary vehicle of respiratory virus transmission is represented by droplet transmission. This is because, in case of coughing or sneezing, small liquid droplets, which may contain an infective amount of virus, are sprayed from the nose or the mouth. Coughs and sneezes are capable to primarily spread droplets of saliva and mucus (droplet transmission); these particles being typically larger than 5 μm. Xie et al. (2007) showed that droplets emitted during coughing with sizes of 60–100 μm fall to the ground within 2 m, while smaller size droplets produced during sneezing can travel more than 6 m.

As already anticipated, airborne transmission through virus-laden PM particles is also possible. This relies on tinier particles, produced by talking or even breathing (Duguid 1946; Papineni and Rosenthal 1997). These particles have a longer atmospheric residence time and can travel further. Exhaled breath particles have sizes and concentrations which depend on respiratory patterns (Morawska et al. 2009). Wan et al. (2014) reported sizes smaller than 5 μm, 80% of which in the range from 0.3 to 1.0 μm. Fairchild and Stampfer (1987) reported observations of particles exhaled during nose and mouth breathing with sizes in the range 0.1–3 μm and a geometric mean concentration of 230 particles per liter during tidal breathing, with 98% of the measured particles having sizes smaller than 1 μm. Papineni and Rosenthal (1997) measured particles exhaled during mouth and nose breathing with sizes between 0.3 and 8.0 μm. They found that more than 84% of these particles are smaller than 1 μm. Furthermore, Morawska et al. (2009) demonstrated that vocalization emits up to an order of magnitude more aerosol particles than breathing, with the number of aerosols increasing with increasing speaking volume (Asadi et al. 2020). Small particles (smaller than 5 μm) may also be produced during coughing and sneezing (Fabian et al. 2011). For example, in the case of influenza, 23% of the particles expelled during coughing were reported to have diameters between 1 and 4 μm and 42% to have diameters smaller than 1 μm (Lindsley et al. 2010). Furthermore, suspended microscopic aerosol particles may also consist of residual solid components of evaporated respiratory droplets, which are even tinier and may remain suspended longer (Asadi et al. 2020).

In the evaluation of the potential role of atmospheric aerosols as virus carriers, the capability of atmospheric particles to incorporate small potentially infectious droplets produced during breathing or speaking has to be properly assessed. The primary process allowing atmospheric particles (PM_2.5_ and PM_10_) to incorporate tiny infectious droplets (smaller than 1 μm) is represented by coagulation. In a coagulation process, two particles collide and eventually adhere, or coalesce, with four fundamental mechanisms potentially determining particles’ collisions: i.e., Brownian diffusion, laminar shear, turbulent fluctuations, and particle differential vertical velocity. As a result of coagulation, a larger particle is created from two smaller particles. Consequently, coagulation leads to a reduction in the number of particles and an increase in their sizes (Smoluchowski 1916, 1918). Eventually, small particles exhaled during breathing or speaking, or even coughing or sneezing, each one carrying a limited virus infective amount, may colloid and coalesce with suspended PM particles, eventually leading to PM particles carrying a sufficient virus amount for the infection to be transmitted. Infected PM particles can remain suspended in the air for hours and travel over much longer distances than the large particles emitted during coughing or sneezing, ultimately entering the human respiratory system.

To obtain a better comprehension of the potential role of aerosol particles in virus transmission, the relationship between virus infectivity and particle size has to be carefully studied (Gralton et al. 2011). Few studies are presently available on this topic. For example, Scott and Sydiskis 1976) reported the presence of a lower infectious dose in smaller size particles (2 μm) than in larger particles (10 μm). Thus, PM_10_ particles can act as a more efficient carrier than PM_2.5_ particles. Furthermore, particle size may also affect virus survivability. In this regard, Tyrrell (1967) demonstrated that rhinovirus survives better in coarse particles (> 4 μm) than in smaller particles (0–4 μm), while Appert et al. (2012) found that adenovirus infectivity is better preserved in coarse particles compared with fine particles. Nevertheless, it is to be underlined that coarse particles primarily deposit in the nasal, pharyngeal and laryngeal passages, and in the trachea, while fine particles are primarily deposited in the respiratory bronchioles and alveoli, with important effects in terms of enhanced virus infectivity.

### Specific remarks for SARS-CoV-2/COVID-19

Atmospheric aerosols were demonstrated to play an important role in triggering pro-inflammation and oxidation processes in the lungs of patients infected with SARS-CoV-2. Prolonged exposure to PM_2.5_ and PM_10_ has been found to induce acute respiratory inflammation and immunological alterations in these patients (Gemmati et al. 2020; Leung et al. 2020). SARS-CoV-2 is more likely to infect weaker and elderly patients exposed to critical environmental conditions, especially those affected by the simultaneous presence of two or more chronic diseases (Guan et al. 2020). Additionally, patients with compromised immune functions have higher chances to be infected than others. High concentrations of PM_2.5_ particles may also lead to an over-expression of the viral receptor ACE-2 in the human respiratory system (see papers by Borro et al. 2020; Gemmati et al. 2020; Devaux et al. 2020; Bunyavanich et al. 2020; Leung et al. 2020). It has been speculated that the presence of an elevated number of viral receptors in the host cells may increase the susceptibility to SARS-CoV-2 infection. Thus, pollution-induced over-expression of ACE-2 on human airways may favor SARS-CoV-2 infectivity.

The presence of SARS-CoV-2 was successfully detected on PM_10_ particles in a polluted area in Northern Italy in the period 21 February–13 March 2020 (Setti et al. 2020b, 2020c), i.e., at the time of the pandemic outbreak. In these research works, the presence of SARS-CoV-2 viral RNA was revealed through the detection of the highly specific “RtDR gene” on 8 filters out of 34 samples in outdoor PM_10_ pollution samples. However, these measurements did not allow to assess whether the virus was viable and its amount was sufficiently high to induce infection. Additionally, only fragments of the viral RNA and not the complete viral RNA sequence were detected. It is to be specified that viruses are much smaller than pollution particles, the former typically ranging from 70 to 90 nm (Kim et al. 2020). At present, there is no definitive, clear, and univocal assessment of the aerosol viral load and the minimum infectious dose necessary to transmit COVID-19, although preliminary ongoing studies on SARS-CoV-2 and previous studies focusing on other viral respiratory pathologies indicated that very low virus loads can initiate infection (Nicas et al. 2005, see Section 4 for more details).

In order to alert the international community on the potential risks of SARS-CoV-2 infection through sneezing and coughing, in March 2020, the World Health Organization issued an official report (WHO 2020a) which advised on the need to maintain a distance of at least 1 m between individuals. However, this recommendation was not taking into account the potential role of atmospheric particles as virus carriers, i.e., airborne transmission. More recently (July 2020), the World Health Organization has acknowledged the emerging evidence that SARS-CoV-2/COVID-19 can be spread by tiny particles suspended in the air, thus recognizing the potential role of airborne transmission in causing infection within the recipient individuals as a result of the inhalation of aerosol particles containing a sufficient virus quantity (WHO 2020b). Airborne transmission has been hypothesized to more likely characterize symptomless infected patients (Rothe et al. 2020; He and Han 2020). Evidence of the importance of airborne transmission in symptomless infected patients was reported for previous respiratory viruses (Fernstrom and Goldblatt 2013). At present, no study is available specifically focusing on the analysis of breath and cough samplings from patients infected with SARS-CoV-2/COVID-19, but SARS-CoV-2 has been detected in the indoor air samples in hospitals (Liu et al. 2020; Santarpia et al. 2020).

The incorporation of viruses by atmospheric particles through coagulation is a process with a high efficiency variability. SARS-CoV-2 stability in aerosols was recently estimated by van Doremalen et al. (2020), who determined virus decay rates using a Bayesian regression model. Results from these authors indicate that SARS-CoV-2 remains viable in aerosols for a duration of ~ 3 h, with an half-life of 1.1 h.

The effectiveness of airborne transmission strongly depends on SARS-CoV-2 infectious dose, which represents the virus amount needed to initiate an infection. The infectious dose is highly variable for different respiratory viruses, ranging from just 10 virus-laden particles—for example, for adenovirus—to thousands for other human viruses (Lakdawala and Gaglia 2020). However, the exact number of SARS-CoV-2 viruses needed to trigger COVID-19 infection has not been determined yet and its determination represents an important topic for scientific investigation. The intense worldwide pandemic outbreak of COVID-19 clearly testifies that SARS-CoV-2 is very contagious, but this may indicate either that a limited number of viruses are needed for infection (low infectious dose) or that infected people release a lot of viruses in the external environment.

## Assessment of the potential role of airborne transmission

In the above sections, we provided a detail overview of the research knowledge and scientific literature on atmospheric particulate pollution and its potential interaction with the human respiratory system. In what follows, the paper methodology is illustrated and developed, which includes a model component, an observational component, and a statistical analysis component.

An analytical model has been developed in order to get a rough quantitative assessment of the potential role of airborne transmission through virus-laden PM particles in conveying SARS-CoV-2 in the human respiratory system and trigger COVID-19. The purpose of the model is to assess this additional transmission vehicle with a specific attention to indoor conditions. A proper simulation of this process requires specific information on exhaled particles’ sizes and concentrations, their residence time, transported viral dose, and minimum infective dose. The model simulates particle collection efficiency, accounting for the combined effect of collision and coagulation efficiencies in the formation of virus-transmitting PM particles. The model provides a quantitative assessment of the role of Brownian diffusion, laminar shear, turbulent fluctuations, and gravitational and drag forces.

Concerning the transported viral dose and the minimum infective dose, only limited virological information is available in the open international literature specifically for SARS-CoV-2. However, for the purpose of getting a rough estimate of the importance of airborne transmission through virus-laden PM_2.5_ and PM_10_ particles, missing specific information on the above quantities for SARS-CoV-2 can be replaced with analogous information from other viral pathologies. This obviously leads to results affected by a large degree of uncertainty.

The developed analytical model relies on the following assumptions/hypotheses.

- Outdoor PM_2.5_ and PM_10_ concentrations are assumed to be 60 and 70 μg/m^3^, respectively; these values are representative of the high particulate pollution conditions observed in Northern Italy in the weeks preceding the pandemic outbreak (values exceeding 60 μg/m^3^ for PM_10_ and PM_2.5_ were observed 8 and 6 times, respectively, during the month of February 2020 in all metropolitan cities in Lombardia, see following Section 6.1). Outdoor PM_2.5_ and PM_10_ concentrations are assumed to be also present indoor. In this regard, it is to be specified that PM_2.5_ and PM_10_ pollution concentrations in outdoor environments are frequently found to be highly correlated with indoor concentrations (Mohammed et al. 2015; Pallarés et al. 2019; Saramak 2019), and are often characterized by comparable concentrations (Massey et al. 2009). Thus, the presence of high outdoor PM_2.5_ and PM_10_ pollution levels is likely to translate into high indoor PM_2.5_ and PM_10_ concentrations, which is what we assume.
- SARS-CoV-2 is assumed to remain viable in aerosols for ~ 3 h in indoor conditions, with a half-life of 1.1 h and a reduction in infectious titer from 10^3.5^ to 10^2.7^ TCID50 per liter of air (van Doremalen et al. 2020).
- The considered indoor space has a volume of 50 m^3^ and includes 5 individuals, all of them but one asymptomatic infected patients.
- 80% of the particles exhaled during breathing or speaking are assumed to have diameters smaller than 1 μm (see above in Section 3.1 in more detail: Wan et al. 2014; Fairchild and Stampfer 1987; Papineni and Rosenthal 1997), while 40% of the particles expelled during coughing are assumed to have diameters smaller than 1 μm (Lindsley et al. 2010).
- The number of particles exhaled on tidal breathing by subjects infected by a respiratory virus is assumed to be 5000 per liter (7200 particles per liter were reported by Fabian et al. (2011) for human rhinovirus (HRV), while 4644 particles per liter were reported by Wan et al. (2014) for mechanically ventilated patients affected by pneumonia); the number of particles produced on coughing by influenza-infected patients was estimated to be 75,400 particles per cough (Lindsley et al. 2012), which is the number assumed in the model.
- Tidal volume of human breath and respiratory rate are assumed to be 500 ml and 15 breaths/min, respectively (Carroll 2007; Riediker and Tsai 2020); thus, 3-h pulmonary ventilation corresponds to a volume of 1350 l; coughing volume and rate are assumed to 250 ml and 2 coughs/min, respectively (Hsu et al. 1994); thus, air expelled during 3-h coughing corresponds to a volume of 90 l.
- The velocity of particles produced on breathing and coughing is assumed to be 1 and 10 m s^−1^, respectively (breathing particle velocities in the range 1–7 m s^−1^ were estimated by Tsuda et al. (2013), while coughing particle velocities in the range 10–30 m s^−1^ and in the range 20–90 m s^−1^ were estimated by Bourouiba (2020) and La Rosa et al. (2013), respectively).

The following equations and additional hypotheses are considered in our simplified model. Small particles exhaled during breathing or speaking and coughing may colloid and coalesce on suspended PM_2.5_ and PM_10_ particles. We assume liquid water to be the predominant component of exhaled particles, while PM_2.5_ and PM_10_ particles are assumed to be primarily carbonaceous/soot particles (density = 1.8–2.1 × 10^3^ kg m^−3^), with a high affinity to water, i.e., a high hygroscopicity (Liu et al. 2013; Henning et al. 2012). Particles are subject to three major classes of motion: uniform motion (primary associated with the gravitational and drag forces), diffusive motion (Brownian diffusion), and the motion of the air mass in which the particle is embedded (wind, turbulence, convective air currents, etc.). The dynamical regime of the suspended particles can be defined through the Knudsen number:

$$ {K}_n=\frac{2\lambda }{d} $$

where λ is the mean free path of the suspending gas and *d* is the diameter of the particle (Baron and Willeke 2001). The free molecular regime characterizes particles which are small compared to the mean free path of the suspending gas, i.e., Kn >> 1 (De Carlo 2004). Particles in this regime tend to follow ballistic streamlines. The continuum regime characterizes particles which are large compared to the mean free path of the suspending gas (Kn << 1, De Carlo 2004), with the gas acting as a continuous fluid flowing round the particle. The mean free path of air molecules is about 0.07 μm (Jennings 1988); thus, the motion of the suspended particles considered in the present study is governed by the continuum regime equations.

In the present continuum regime, the number of colliding aerosol particles is given by the expression (Morris 2002; Friedlander 1977):

1 $$ {N}_{ab}=\alpha \times \beta \left(a,b\right)\times {n}_a\times {n}_b $$

with *n*_*a*_ and *n*_*b*_ being the concentrations of the two classes of colliding particles *a* and *b*, *α* being the collision or attachment efficiency, and *β(a,b)* being the collision frequency in the continuum regime. The collision efficiency is defined as (Phan-Cong and Dinh-Van 1973):

2 $$ \propto ={\left(\frac{x}{r_a+{r}_b}\right)}^2 $$

with *x* being the largest initial horizontal separation of the falling droplet centers and *r*_*a/b*_ being the radii of the two colliding particles. The collision efficiency is taken equal to 1.

The collision frequency includes four distinct terms associated with four distinct processes: the Brownian motion, the fluid laminar and turbulent shears, and the differential settling. The Brownian motion term *β*_*B*_(*a,b*) of the collision frequency can be determined through the expression (Morris 2002):

3 $$ {\beta}_B\left(a,b\right)=4\pi \left({r}_a+{r}_b\right)\left({D}_a+{D}_b\right) $$

with *D*_*a*_ *+ D*_*b*_ being the effective diffusion coefficient between particles. Diffusion coefficients can be determined through the Stokes-Einstein relation (Seinfeld and Pandis 1998):

4 $$ {D}_{a/b}=\frac{kTCc}{6\pi {\mu r}_{a/b}} $$

with *k* being the Boltzmann constant (1.38 J K^−1^), *C*_*c*_ being the slip correction factor (Seinfeld and Pandis 1998), and *μ* being the viscosity of air (1.8 × 10^−5^ kg m^−1^ s^−1^). In air at 293 K and 1 atm, *D* = 1.29 × 10^−11^ for 0.1-μm radius particles, *D* = 5.05 × 10^−12^ for 1-μm radius particles, *D* = 4.92 × 10^−12^ for 2.5-μm radius particles, and *D* = 1.20 × 10^−12^ for 10-μm radius particles.

The laminar fluid shear term *β*_*LS*_(*a,b*) of the collision frequency can be expressed as (Stumm 1992):

5 $$ {\beta}_{LS}\left(a,b\right)=1.33G{\left({r}_a+{r}_b\right)}^3 $$

with *G* being the mean velocity gradient. Values of *G* in the particle size interval of interest for our computations (0.1 μm ≤ r ≤ 5 μm) vary in the range 0.46–10 s^−1^ (Pruppacher and Klett 1997). The turbulent fluid shear term *β*_*TS*_(*a,b*) of the collision frequency can be expressed as (Pruppacher and Klett 1997):

6 $$ {\beta}_{TS}\left(a,b\right)=1.3\mu {\left({r}_a+{r}_b\right)}^3 $$

with *μ* being the turbulent shear rate. Typical values of *μ* are in the range 4–28 s^−1^ (Ackerman 1967). Finally, the differential sedimentation term *β*_*S*_(*a,b*) of the collision frequency can be expressed as (Findheisen 1939):

7 $$ {\beta}_S\left(a,b\right)=\pi {\left({r}_a+{r}_b\right)}^2\left|{v}_a-{v}_b\right| $$

with *v*_*a*_ and *v*_*b*_ being the terminal settling velocities of the two classes of colliding particles *a* and *b*. As a result of gravitational settling, in combination with drag, particles reach a constant velocity *v*_*t*_, called “terminal settling velocity.” The time *τ* required for a particle to reach its terminal settling velocity varies as a function of the particle size, being around 8 × 10^−5^ s for PM_2.5_ particles and around 1.2 × 10^−3^ s for PM_10_ particles (Seinfeld and Pandis 1998). The *terminal settling velocity* can be determined through the expression:

$$ {v}_{a/b}=\frac{r^2\left(\rho -{\rho}_w\right){gC}_c}{9\mu } $$

with *r* being the particle radius; *ρ* being the PM particle density (1.27–1.78 × 10^3^ kg m^−3^ for black carbon, 1.8–2.1 × 10^3^ kg m^−3^ for soot); *ρ*_*w*_ being the density of the particles exhaled when breathing, speaking, coughing, and sneezing (assumed to be composed of liquid water, 0.997 × 10^3^ kg m^−3^); *g* being the gravity acceleration (9.8 m s^−1^); *C*_*c*_ being the slip correction factor (with values varying around 1 in the considered particle size range, Seinfeld and Pandis 1998); and *μ* being the viscosity of air (1.8 × 10^−5^ kg m^−1^ s^−1^). PM_2.5_ and PM_10_ particles have larger *terminal settling velocities* than particles exhaled while breathing, speaking, and part of those emitted when coughing. As a result of this differential velocity, PM_2.5_ and PM_10_ particles eventually collide with exhaled particles. The above expression is applicable to motions characterized by Reynolds numbers Re < 0.1 or particles smaller than about 20 μm (Seinfeld and Pandis 1998).

Expression (6) has also been extended to the coalescence process associated with the primarily horizontal component of the initial part of the motion of the exhaled breathing and coughing particles; this motion is treated as a uniform motion as the one associated with gravitational settling. “Horizontal” coalescence was found to play an important role also in other processes commonly considered to be dominated by “vertical” coalescence, as those associated with convective cloud formation (Khain and Pinsky 1995).

**Fig. 1:**
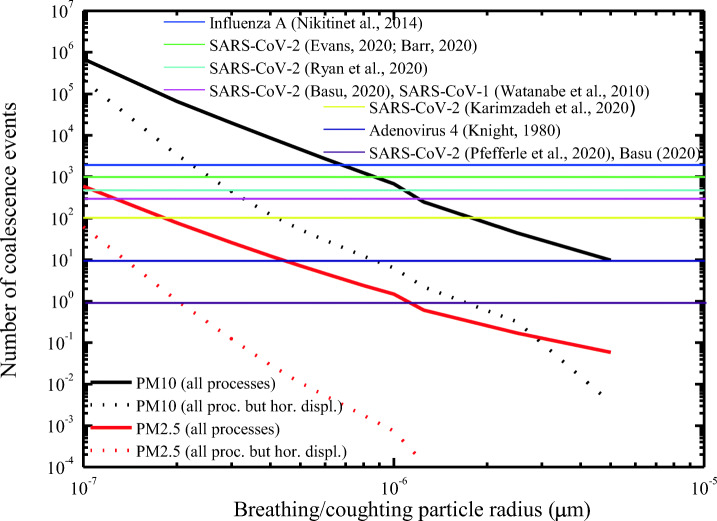
Overall number of coalescing events of breathing and coughing particles onto PM_2.5_ (red lines) and PM_10_ (black lines) particles as a function of the size of the breathing and coughing particles. Two lines are present for each particle size: one (solid line) representing the overall number of coalescing events associated with all considered coagulation processes, the other one (dotted line) representing the number of coalescing events when excluding the coagulation process associated with the primarily horizontal component of the initial motion of exhaled particles. The horizontal lines in the figure represent the minimum infectious doses of SARS-CoV-2 and other respiratory viruses

Based on the above analytical expressions, which describe the different microphysical processes involving PM_2.5_ and PM_10_ particles and the small particles emitted during breathing, speaking, or coughing, it is possible to determine the number of events associated with each coalescence process. This number is highly variable in dependence of the considered process and the size of the breathing and coughing particles colliding with PM_2.5_ and PM_10_ particles. The overall number of coalescence processes of breathing and coughing particles on PM_2.5_ and PM_10_ particles is illustrated in Fig. 1. The overall number of coalescence processes is highly dependent on the size of the breathing and coughing particles. As the contribution of the different processes may strongly vary as a function of the sizes of breathing and coughing particles, both axes in the figure are represented in log-scale. Figure 1 includes two lines for each particle type: one line represents the overall number of coalescence events associated with all the abovementioned coalescence processes, including the one associated with the horizontal component of the initial motion of exhaled breathing and coughing particles; the other line represents the number of coalescing events associated only with Brownian motion, fluid laminar and turbulent shear, and differential settling. Both lines have been reported as in fact the contribution associated with the initial horizontal motion of exhaled breathing and coughing particles appears to be predominant with respect to the other processes, and consequently, results from the model need to be carefully discussed accounting for these differences.

Results in Fig. 1 clearly reveal that the number of coalescence events strongly decreases with the increasing size of breathing/coughing particles. Specifically, for PM_10_ particles, the number decreases from 7 × 10^5^ to 6.5 × 10^4^, 4.5 × 10^3^, 7 × 10^2^, 80 and 10 for breathing/coughing particle sizes increasing from 0.1 to 0.2, 0.5, 1, 2, and 5 μm, respectively. When uniform horizontal coalescence is disregarded, the number of breathing and coughing particles colliding on PM_10_ particles decreases from 2.5 × 10^5^ to 3.5 × 10^3^, 45, 6, 0.6, and 4 × 10^−3^ for breathing/coughing particle sizes increasing from 0.1 to 0.2, 0.5, 1, 2, and 5 μm, respectively. For PM_2.5_ particles, the number of coalescing events is approximately 3 orders of magnitude smaller than for PM_10_ particles. Specifically, the number of coalescence events decreases from 7 × 10^2^ to 80, 7, 1.5, 0.25, and 6 × 10^−2^ for breathing/coughing particle sizes increasing from 0.1 to 0.2, 0.5, 1, 2, and 5 μm, respectively, while when removing uniform horizontal coalescence, the number of coalescence events decreases from 65 to 1.2, 1.2 × 10^−2^, and 7 × 10^−4^ for breathing/coughing particle sizes increasing from 0.1 to 0.2, 0.5, and 1 μm.

The minimum infectious dose of viable SARS-CoV-2 required to cause infection is not exactly known yet, but there are a number of estimates, together with information extracted from studies on other respiratory viruses. Specifically, the minimum infectious dose was estimated by Evans (2020) and Barr (2020) to be ~ 1000, while Ryan et al. (2020) estimated a minimum number of 500 infectious particles to be sufficient to activate infection. A value of 300 is suggested by Basu (2020), while a value of 100 was conjectured by Karimzadeh et al. (2020)*.* Pfefferle et al. (2020) and Basu (2020) hypothesized that even a single viral particle can be sufficient to initiate SARS-CoV-2 infection. Other respiratory viruses were found to be characterized by higher or lower infectivity levels. Specifically, Nikitin et al. (2014) suggested a value of 1950 for influenza A, Watanabe et al. (2010) estimated a minimum infectious dose value of 280 virus particles for SARS-CoV-1, while adenovirus type 4 was found to approach an infectivity of 10 virus particles or less (Knight 1980). All these different values are reported in Fig. 1 as horizontal lines. The figure clearly reveals that the number of coalescing events of breathing/coughing particles onto PM_2.5_ and PM_10_ particles is exceeding most of the infectious dose values indicated above over a large size range for the colliding breathing/coughing particles. This is particularly true for PM_10_ particles, where the number of coalescing events is approximately 3 orders of magnitude higher than for PM_2.5_ particles. Specifically, the number of breathing and coughing particles coalescing on PM_10_ particles exceeds the highest SARS-CoV-2 infectious dose value (1000) for breathing/coughing particle sizes up to 0.9 μm, while the number of breathing/coughing particles coalescing on PM10 particles exceeds the SARS-CoV-2 infectious dose value of 10 for breathing/coughing particles over the size interval 0.1 μm ≤ r ≤ 5 μm. In case the uniform horizontal coalescence mechanism is ignored, the number of breathing and coughing particles coalescing on PM_10_ particles exceeds the highest SARS-CoV-2 infectious dose value for breathing/coughing particle sizes up to 0.15 μm, while the number of coalescing events exceeds the lowest SARS-CoV-2 infectious dose value (1) for breathing/coughing particle sizes up to 2 μm. Finally, the number of breathing and coughing particles coalescing on PM_2.5_ particles exceeds the minimum infectious doses of 100, and 10 and 1 for breathing/coughing particle sizes up to 0.18, 0.45, and 1.2 μm, respectively.

The above results assume that all breathing and coughing particle coalescing on PM_2.5_ and PM_10_ particles carry at least one virion, i.e., a complete viral particle. This is obviously a very uncertain assumption as in fact this information is not specifically available for SARS-CoV-2. The number of virions carried by suspended particles is strongly dependent on their size, the value increasing with increasing size. An indirect estimate of this quantity can be obtained from the data available on the number of virus copies present in SARS-CoV-2 sputum samples, with a value of 2.35 × 10^9^ copies per milliliter reported by Wölfel et al. (2020) and a value up to 1.34 × 10^11^ copies per milliliter reported by Pan et al. (2020). Considering the same virus concentration in the sputum and in the exhaled particles, the number of virions carried by each suspended particle is in the range 0.0012–0.0702 for a particle radius of 0.5 μm, in the range 0.0098–0.56 for a particle radius of 1 μm, in the range 0.079–4.49 for a particle radius of 2 μm, and in the range 1.23–70.16 for a particle radius of 5 μm. These numbers confirm that airborne transmission through virus-laden PM particles represents a potentially important vehicle for virus transmission in particulate pollution conditions which may effectively contribute to the infectious spread.

All in all, the results from the present analytical model, in combination with measurements of PM_2.5_ and PM_10_ particulate pollution, and virological and epidemiologic information from literature papers, allow establishing the important potential role of airborne transmission in conveying a contagious virus amount in the respiratory system. This transmission mechanism is primarily associated with the small particles emitted during breathing, speaking, and partially also during coughing and sneezing, each carrying a limited virus infective amount, which may colloid and coalesce with suspended PM particles, eventually leading to PM particles carrying a sufficient virus amount for the infection to be transmitted.

While evidence of the presence and viability of SARS-CoV-2 and other virus particles has been reported in the literature for laboratory-controlled indoor conditions (van Doremalen et al. 2020), no information is available on the effect of variable temperature, humidity, UV radiation, atmospheric stability, transport conditions, and atmospheric oxidant capacities in atmospheric outdoor environments (Lolli et al. 2020). This implies that airborne transmission through virus-transmitting PM particles, while having a more uncertain role in outdoor conditions, is likely to be important in indoor environments.

Finally, while airborne transmission through virus-laden PM particles represents a potentially important vehicle for virus transmission in particulate pollution conditions, it is necessary to emphasize that, even in unpolluted conditions, in unventilated indoor spaces with a number of infected individuals, especially asymptomatic ones, the simple ingestion of briefing and coughing particles exhaled by these individuals may represent an important vehicle for virus transmission.

## Air quality datasets

PM_10_ measurements considered in this paper for comparison with epidemiologic parameters are from the Italian ground-based network of air quality monitoring stations which is run by different regional and provincial environment agencies. Filter-based samplers of PM_2.5_ and PM_10_ particles, equipped with size-selective inlets capable to discriminate particles smaller than 2.5 and 10 μm, respectively, represent the primary approach to monitor atmospheric particulate matter in the ground network of air quality monitoring stations. Data within this network are collected in a coordinated manner based on a well-established common procedure. Consequently, differences between measurements from different sensors to be attributed to sensors’ biases are assumed to be negligible.

The effective impact of PM_2.5_ and PM_10_ particles on COVID-19 infection outbreak strongly depends on particle persistency in the air, i.e., on the duration of the exposure of the human respiratory system to particulate pollution throughout the day. However, in most cases, gravimetric measurements are based on the analysis of filters collecting particulate material over the duration of the day, and, consequently, lack in temporal resolution. Thus, ground PM measurements are scarcely usable when particle concentration variability during a specific day or at specific times of the day needs to be inferred. Alternative measurement techniques or datasets have then to be considered to eventually assess the actual duration of the human respiratory system pollution exposure throughout the day. Particularly effective in this direction is the use of PM_2.5_ and PM_10_ data from near-real-time ECMWF-CAMS analysis, which are provided with hourly resolution and a grid size of 10 × 10 km. CAMS near-real-time reanalysis, used in this research effort, is the most recent global reanalysis of atmospheric composition and air quality data, which is produced by the Copernicus Atmosphere Monitoring Service (CAMS) of the European Centre for Medium-Range Weather Forecasts (ECMWF). PM_2.5_ and PM_10_ data from ECMWF-CAMS near-real-time reanalysis is based on the reanalyses from a number of state-of-the-art numerical air quality models developed in Europe. These models, each one relying on its own data assimilation system, perform daily retrospective analyses of pollutants near the surface by assimilating 1-day-old observations. Surface observations, collected on a daily basis from the European Environmental Agency (EEA), are the main source of assimilated data. The analyses from all partner models are combined via an ensemble approach, consisting in calculating the median value of the individual outputs. ECMWF-CAMS near-real-time reanalysis data, while characterized by a high time (1 h) and horizontal (10 × 10 km) resolutions, may result less accurate than the co-located simultaneous ground-based in situ measurements at the exact location of the ground station as a consequence of its lower horizontal resolution.

In the next section, geo-located total column and tropospheric NO_2_ and HCHO column measurements are also reported. These measurements are collected by the satellite sensor TROPOMI onboard Copernicus Sentinel-5P. TROPOMI is a non-scanning nadir-viewing, passive grating imaging spectrometer, with swath width of 2600 km and a spatial sampling of 7 × 7 km, covering wavelength bands between the ultraviolet and the shortwave infrared. The satellite is located on a near-polar, sun-synchronous orbit, with high inclination (approximately 98.7°) at an altitude of approximately 824 km, with nadir overpasses at 13:30 local time (ascending node crossing time). The satellite performs 14 orbits per day, 227 orbits per cycle, and its orbital cycle, i.e., the time taken for the satellite to pass over the same geographical point on the ground, is 16 days. In addition to NO_2_ and HCHO, TROPOMI also measures O_3_, SO_2_, CO, and CH_4_; all datasets are provided with a grid size of 3.5 × 3.5 km. Observations of NO_2_ and HCHO from TROPOMI are illustrated in this paper with the only purpose of confirming their presence during the large particulate pollution outbreak events observed in mid-February 2020 and arguing on their potential role in secondary aerosol formation. In this regard, it is specified that, while the conversion of NO_2_ into particulate phase nitrate is a well-understood physicochemical process, characterized by reasonably well-known conversion rates, volatile organic compounds, as formaldehyde (HCHO), undergo atmospheric degradation processes generating oxidized products, which ultimately may or may not contribute to secondary organic aerosol formation. Formaldehyde may contribute to O_3_ pollution through photochemical reactions (Carter 1994; Russell et al. 1995), which favor the formation of secondary organic aerosol via the provision of OH radicals (Yang et al. 2018). Atmospheric formaldehyde is a product of isoprene oxidation (Palmer et al. 2003) and isoprene emitted by vegetation is an important precursor of secondary organic aerosols (Marais et al. 2016). Direct HCHO emission, closely related to CO emission, takes also place as a result of incomplete combustion processes.

## Results

### Evolution of PM_2.5_ and PM_10_ concentrations throughout the month of February 2020

**Fig. 2:**
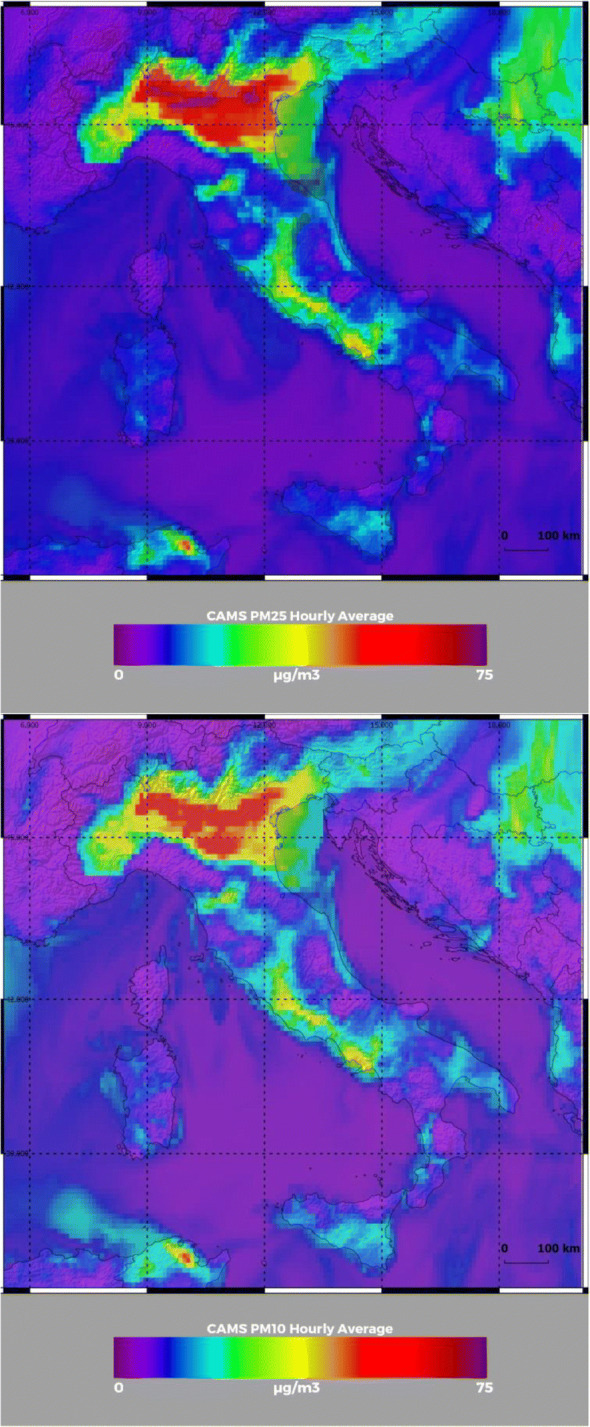
PM_2.5_ (upper panel) and PM_10_ (lower panel) concentrations at 00:00 UTC on 17 February 2020 from near-real-time ECMWF-CAMS analysis over an area encompassing the Italian peninsula and portions of the surrounding nations

**Fig. 3:**
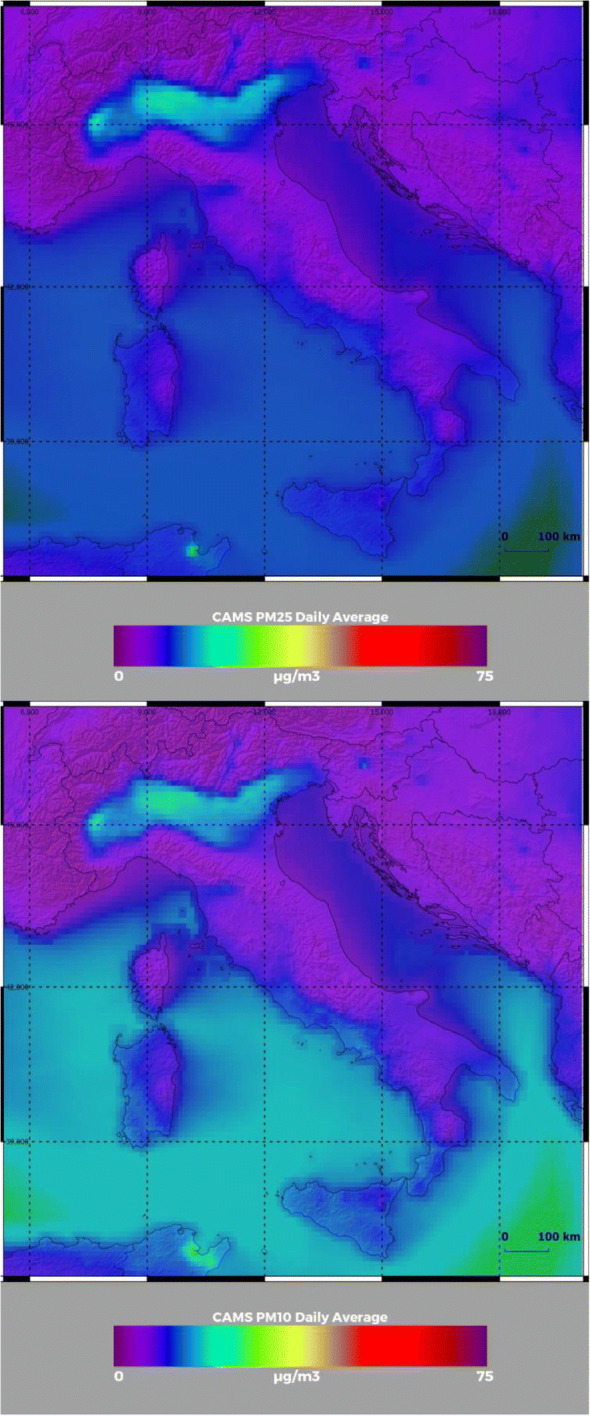
PM_2.5_ (upper panel) and PM_10_ (lower panel) concentrations at 00:00 UTC on 29 February 2020 from near-real-time ECMWF-CAMS analysis over an area encompassing the Italian peninsula and portions of the surrounding nations

Figure 2 shows PM_2.5_ and PM_10_ concentrations at 00:00 UTC on 17 February 2020, as provided by the near-real-time ECMWF-CAMS analysis. The figure covers an area encompassing the Italian peninsula and portions of the surrounding nations. This figure represents the pollution situation in the central day of the second of the three major particulate matter pollution outbreaks (15–19 February 2020) which preceded the pandemic onset. The figure clearly reveals the presence of enhanced PM_2.5_ and PM_10_ concentrations over large portions of the Po Valley, with levels exceeding 60 μg/m^3^ for both species. These values are largely exceeding the limits of 25 μg/m^3^ for PM_2.5_ and 50 μg/m^3^ for PM_10_ defined by the WHO Air quality guideline (WHO 2005). This figure provides a snapshot of the highly polluted conditions present in the Po Valley shortly before (1–2 weeks) the pandemic outbreak. Figure 3 shows PM_2.5_ and PM_10_ concentrations at 00:00 UTC on 29 February 2020, again from the near-real-time ECMWF-CAMS analysis. This figure provides a snapshot of the pollution situation few days after the shutdown of all vehicular and industrial activities associated with the lockdown in Northern Italy on 25 February 2020. The selected date (29 February 2020) is intended to be indicative of the low PM concentration conditions found on a number of consecutive days in the final part of February 2020. PM_2.5_ and PM_10_ concentrations are found to have dropped abruptly, with levels not exceeding 15–20 μg/m^3^ for both species.

**Fig. 4:**
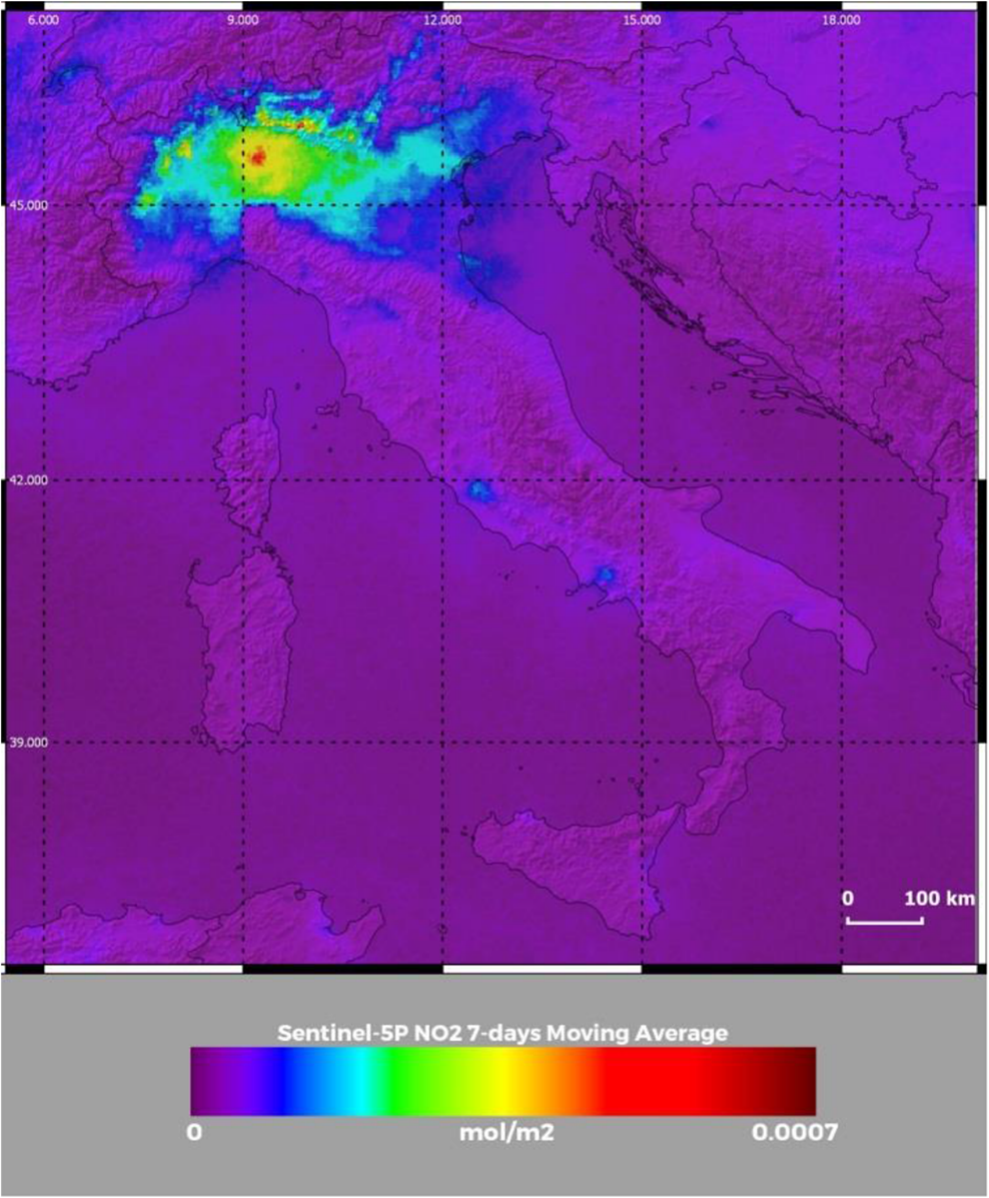
NO_2_ concentrations at 12:30 UTC on 17 February 2020 from Copernicus Sentinel-5P TROPOMI data over an area encompassing the Italian peninsula and portions of the surrounding nations. Data are obtained as 7-day averages centered on the reference day, i.e., on 17 February 2020, thus including the data from 14 to 20 February 2020

**Fig. 5:**
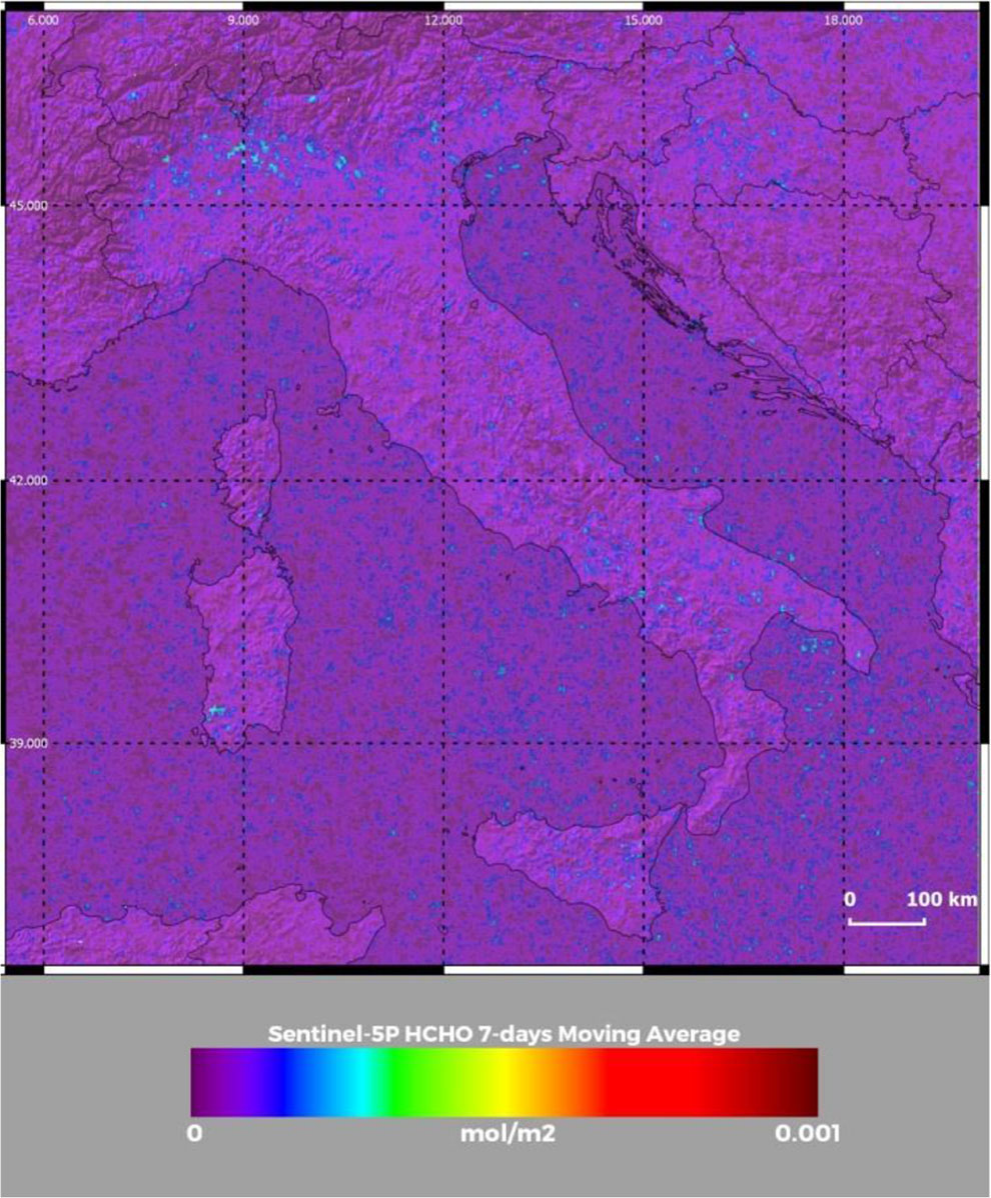
HCHO (formaldehyde) concentrations at 12:30 UTC on 17 February 2020 from Copernicous Sentinel-5P TROPOMI data over an area encompassing the Italian peninsula and portions of the surrounding nations. Data are obtained as 7-day averages centered on the reference day, i.e., on 17 February 2020, thus including the data from 14 to 20 February 2020

Figure 4 illustrates the NO_2_ concentrations from TROPOMI onboard the Copernicus Sentinel-5P at 12:30 UTC on 17 February 2020 for the same area considered in Figs. 2 and 3. The figure reveals the presence of peak NO_2_ values over the metropolitan area of Milan (5 × 10^−4^ mol m^−2^), but high values, in excess of 1.5–2 × 10^−4^ mol m^−2^, are also observed over large portions of the Po Valley. Peak values of lower amplitude (~ 1.5 × 10^−4^ mol m^−2^) are also found over the metropolitan areas of Rome and Napoli. These measurements testify the important potential contribution of secondary aerosol formation in the observed PM_2.5_ and PM_10_ pollution event. Figure 5 illustrates the HCH (formaldehyde) concentrations from TROPOMI at the same time and over the same geographical area considered in Figs. 2, 3, and 4. Values around 2.5 × 10^−4^ mol m^−2^ are found in the upper portion of the Po Valley, in coincidence with the high PM_2.5_ and PM_10_ concentrations, thus further supporting the hypothesis of the potential role played by secondary aerosol formation. Due to the low signal-to-noise ratios of NO_2_ and HCHO concentration measurements, data in Figs. 4 and 5 are 7-day averages centered on the reference day, i.e., on 17 February 2020, thus including the data from 14 to 20 February 2020.

Measurements collected over the period January–February 2020 by the ground-based network of air quality monitoring stations (not shown here) reveal that threshold levels for PM_10_ (< 50 μg/m^3^) were exceeded on 20–35 days in large portions of the Po Valley, with levels in excess of 80 μg/m^3^ occasionally observed in the 1–3 weeks preceding the contagious activation around 25 February 2020. Threshold values for PM_2.5_ indicted in WHO air quality guidelines (< 25 μg/m^3^) were exceeded on more than 40 days over the period January–February 2020 in large portions of the Po Valley (again not shown here), with levels up to 70 μg/m^3^ observed in the weeks preceding the contagious activation.

**Fig. 6:**
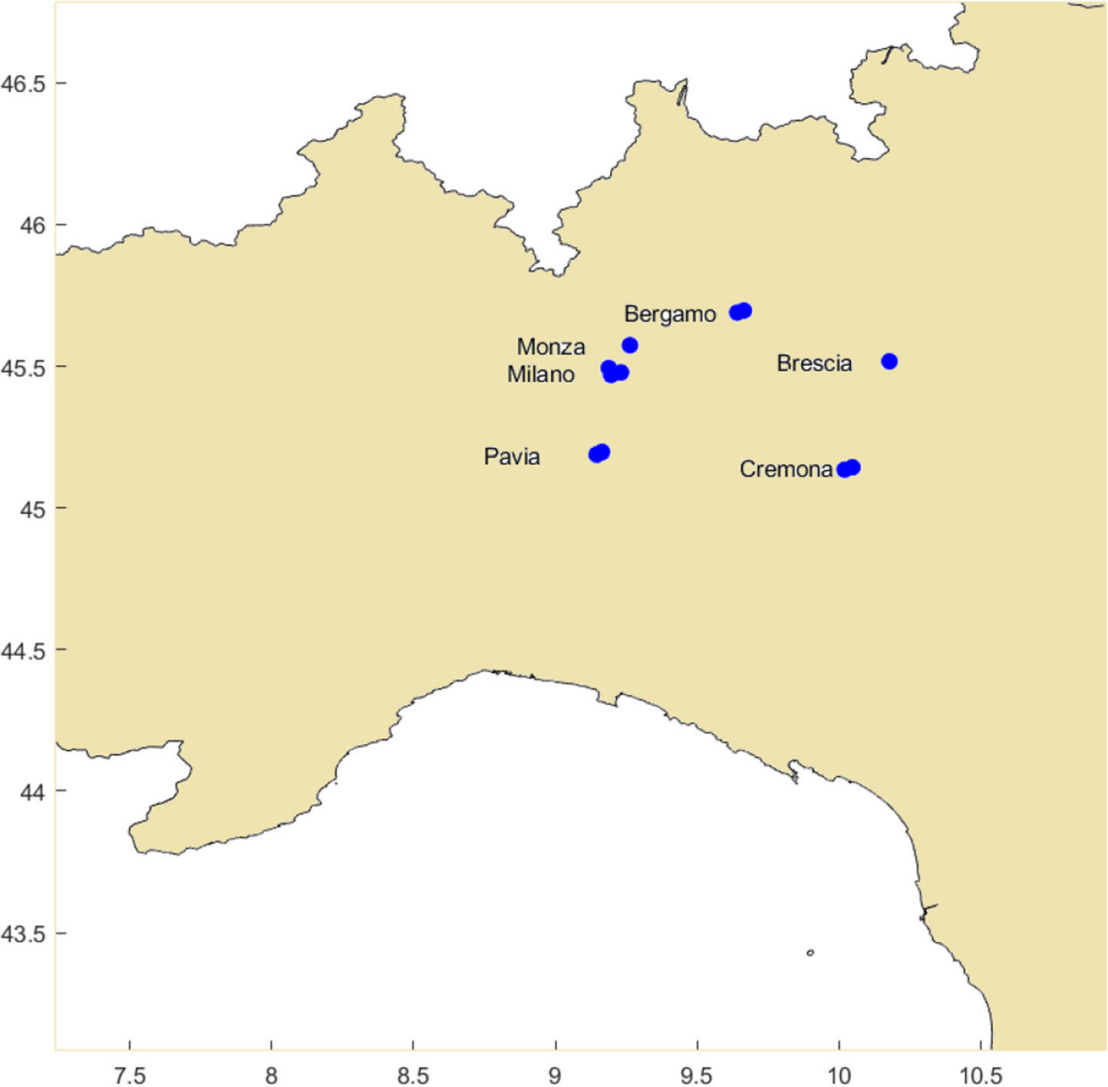
Locations of the metropolitan cities in Lombardia (Bergamo, Brescia, Cremona, Milano, Monza, and Pavia), whose data are considered in Figs. 6 and 7. The locations of one to three stations are considered for each city

**Fig. 7:**
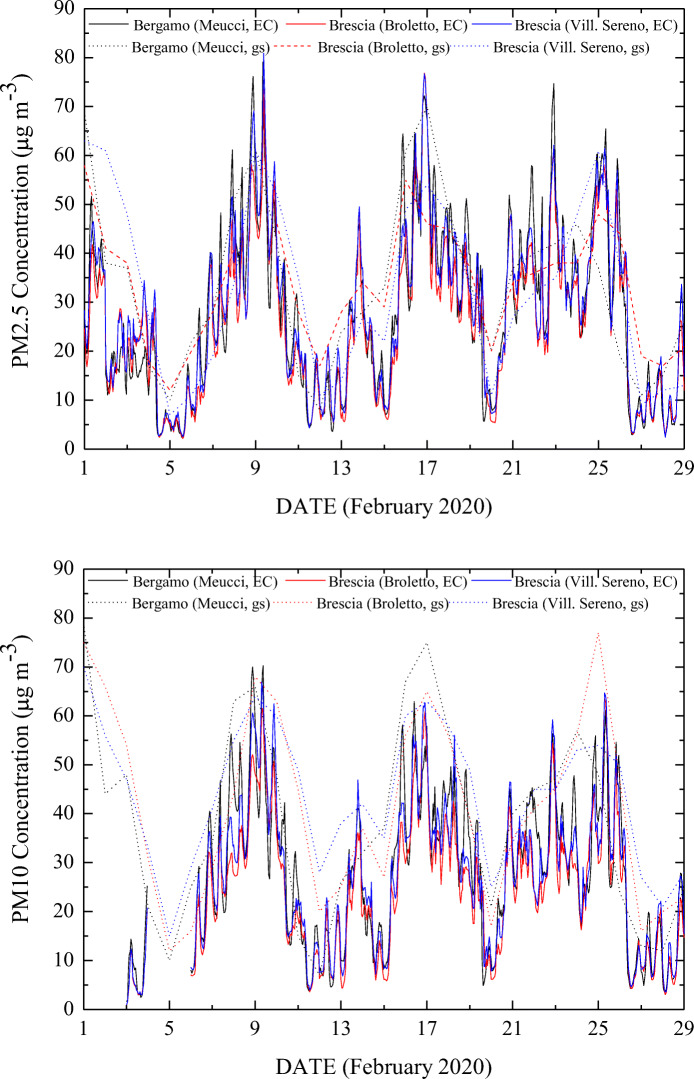
PM_2.5_ (upper panel) and PM_10_ (lower panel) concentrations over the month of February 2020 as measured by the three ground stations (gs), one in Bergamo (via Meucci, 45° 41′ 24″ N, 09° 38′ 28″ E) and two in Brescia (via Broletto, 45° 32′ 23 ″ N, 10° 13′ 24″ E; Villaggio Sereno, 45° 31′ 04″ N, 10° 10′ 41″ E), together with the data from near-real-time ECMWF-CAMS analysis (EC)

The variability of PM_2.5_ and PM_10_ concentrations over the month of February 2020 for several metropolitan cities in Lombardia (Bergamo, Brescia, Cremona, Milano, Monza, and Pavia, see locations in the map in Fig. 6) has been considered. Specifically, Fig. 7 illustrates PM_2.5_ and PM_10_ concentration measurements during the month of February 2020 from three ground-based stations, one in Bergamo (via Meucci, 45° 41′ 24″ N, 09° 38′ 28″ E) and two in Brescia (via Broletto, 45° 32′ 23″ N, 10° 13′ 24″ E; Villaggio Sereno, 45° 31′ 04″ N, 10° 10′ 41″ E), included in the network of air quality monitoring stations run by the regional and provincial environment agencies. In the figure, these measurements are compared with the corresponding data from the hourly near-real-time ECMWF-CAMS analysis. ECMWF-CAMS analysis well reproduces PM_2.5_ and PM_10_ concentration values measured by the ground-based stations, thus revealing its capability to capture the high-resolution variability of PM_2.5_ and PM_10_ concentrations within each single day in this period. This comparison clearly highlights that very high peak concentration values (up to ~ 80 μg/m^3^) are occasionally observed at specific times of the day, such peaks not being detected in the daily-averaged ground station measurements.

**Fig. 8:**
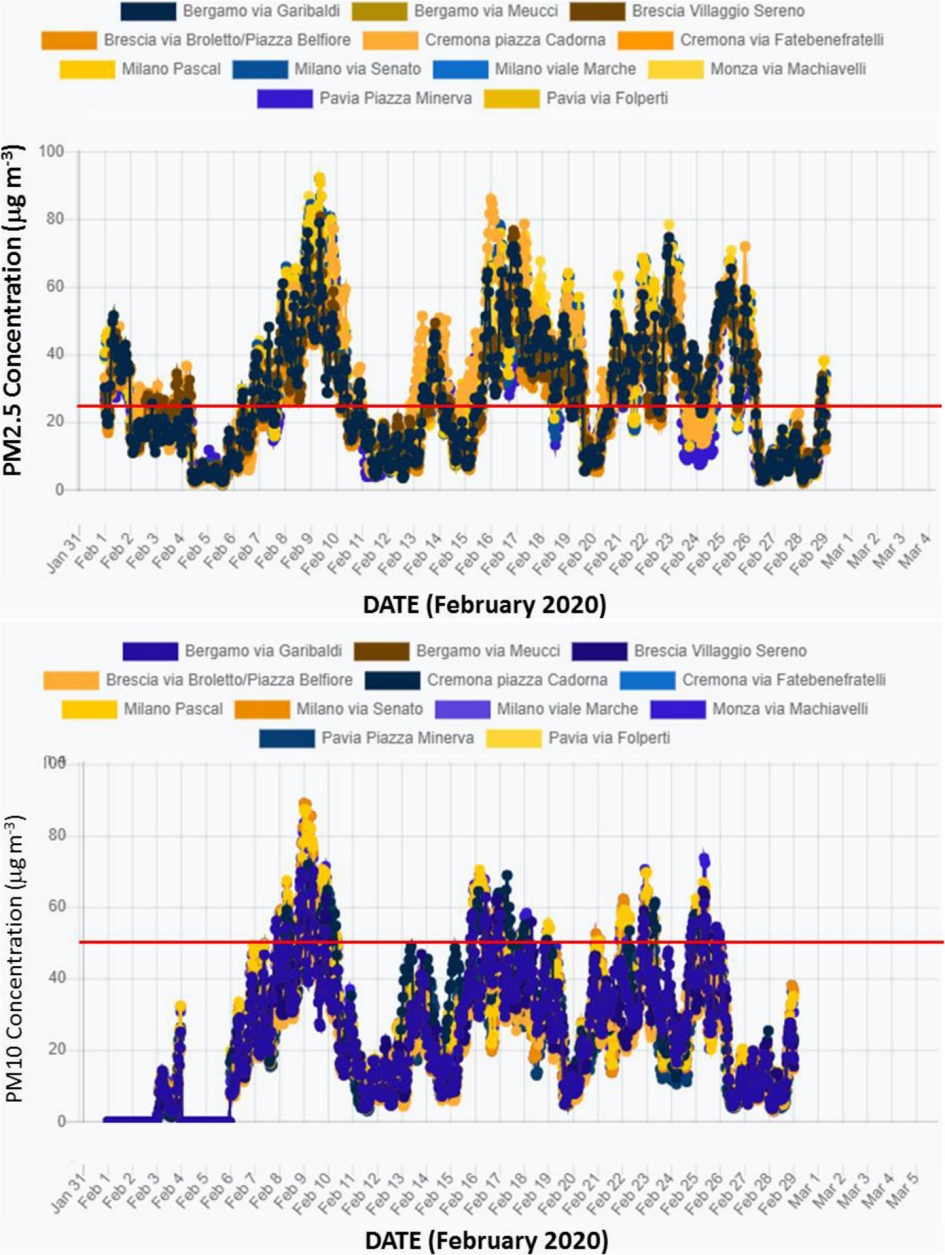
Near-real-time ECMWF-CAMS analysis of PM_2.5_ (upper panel) and PM_10_ (lower panel) concentrations over the month of February 2020 for several metropolitan cities in Lombardia (Bergamo, Brescia, Cremona, Milano, Monza, and Pavia). One to three locations are considered for each city

Figure 8 illustrates the variability of PM_2.5_ and PM_10_ concentrations over the month of February 2020 for several metropolitan cities in Lombardia (Bergamo, Brescia, Cremona, Milano, Monza, and Pavia) as obtained from near-real-time ECMWF-CAMS analysis. Several air quality monitoring stations are considered, when available, for each city. The figure highlights the high PM_2.5_ and PM_10_ peak values present during the month of February 2020 in all considered cities, with values in excess of 60 μg/m^3^ observed 3 to 9 times for PM_2.5_ and 3 to 14 times for PM_10_. This figure also reveals that three major particulate matter pollution outbreak events took place during the month of February 2020: the first one covering the period 06–11 February, the second one covering the period 15–19 February, and the third one covering the period 20–26 February.

In the present research effort, for the purpose of comparing PM_10_ measurements with epidemiologic parameters, we focused our attention on the PM_10_ pollution levels observed over the 12-day period from 15 to 26 February 2020, when very high and persistent PM_10_ concentration values were present over a major portion of the Po Valley.

### Correlations between epidemiologic parameters and PM concentrations

The effective impact of PM_2.5_ and PM_10_ particles on SARS-CoV-2/COVID-19 infection outbreak is expected to be strongly dependent on particle persistency in the air, i.e., on the duration of the effective exposure to particulate pollution of the human respiratory system throughout the weeks preceding the pandemic onset. In a previous paper by Borro et al. (2020), the variability of the infection rate, the mortality rate, and the case fatality rate as a function of particle concentration was estimated for PM_2.5_ particles only, recognizing a primary role of these particles in inducing an over-expression of ACE-2 in the human respiratory system (see papers by Gemmati et al. 2020; Devaux et al. 2020; Bunyavanich et al. 2020; Leung et al. 2020). In the present paper, we extend the analysis to PM_10_ particles, thus assessing the incidence of this additional pollution source on the observed epidemiologic parameters. Specifically, PM_10_ concentration measurements over the period 15–26 February 2020 are compared with epidemiologic data for all 110 Italian provinces, as reported by the Italian Statistics Institute (ISTAT 2020), over the period 20 February–31 March 2020. The list of all 110 Italian provinces, their population, and their population density is reported in Table 1. Actually, the epidemiologic data report from ISTAT includes only 107 provinces, as in fact four provinces in Southern Sardinia (Carbonia-Iglesias, Ogliastra, Olbia-Tempio, and Medio Campidano) are grouped together as “Sud Sardegna.”

**Table 1:** List of all 110 Italian provinces, their population, and their population density. Four provinces in Southern Sardinia (Carbonia-Iglesias, Ogliastra, Olbia-Tempio, and Medio Campidano) are grouped together as “Sud Sardegna”

*N*.	Province	Population (inhabitants)	Density (inhab/km^2^)	*N*.	Province	Population (inhabitants)	Density (inhab/km^2^)
1	Agrigento	442,049	120.0477	56	Messina	636,653	48.9802
2	Alessandria	426,658	241.3716	57	Milano	3,218,201	276.2157
3	Ancona	474,124	38.8868	58	Modena	700,862	168.1950
4	Aosta	126,883	144.0697	59	Monza	868,859	298.5784
5	Arezzo	344,374	57.1857	60	Napoli	3,107,006	672.4241
6	Ascoli Piceno	209,450	395.5944	61	Novara	370,143	69.7781
7	Asti	216,677	199.4911	62	Nuoro	156,096	322.8946
8	Avellino	423,506	263.6840	63	Oristano	160,746	253.8787
9	Bari	1,260,142	68.2099	64	Padova	936,274	215.3552
10	Barletta	392,546	258.7006	65	Palermo	1,268,217	89.3426
11	Belluno	205,781	458.7316	66	Parma	448,899	286.5306
12	Benevento	279,675	201.9944	67	Pavia	547,251	236.9538
13	Bergamo	1,109,933	164.2861	68	Perugia	660,690	146.3796
14	Biella	178,551	185.7507	69	Pesaro e Urbino	360,711	127.7137
15	Bologna	1,009,210	412.8634	70	Pescara	321,309	301.8640
16	Bolzano/Bozen	524,256	285.7695	71	Piacenza	286,758	67.3673
17	Brescia	1,262,318	218.9951	72	Pisa	421,851	248.2561
18	Brindisi	397,083	174.3952	73	Pistoia	291,839	189.3557
19	Cagliari	560,373	172.9056	74	Pordenone	312,051	188.2805
20	Caltanissetta	269,710	1928.3570	75	Potenza	370,680	174.0820
21	Campobasso	224,644	255.1238	76	Prato	254,608	198.9591
22	Caserta	924,166	2072.276	77	Ragusa	321,359	727.7163
23	Catania	1,113,303	272.7475	78	Ravenna	391,414	134.1421
24	Catanzaro	362,343	429.7090	79	Reggio Calabria	553,861	171.3036
25	Chieti	389,169	123.9844	80	Reggio Emilia	532,483	202.6700
26	Como	600,190	180.4939	81	Rieti	157,420	300.0037
27	Cosenza	711,739	141.2050	82	Rimini	336,786	59.1065
28	Cremona	359,388	110.0664	83	Roma	4,353,738	114.9937
29	Crotone	175,566	225.7783	84	Rovigo	238,588	57.5187
30	Cuneo	589,108	372.0382	85	Salerno	1,104,731	255.7531
31	Enna	168,052	56.57933	86	Sassari	333,116	136.5952
32	Fermo	174,849	329.2485	87	Savona	279,408	57.3111
33	Ferrara	348,362	84.5532	88	Siena	268,341	133.2064
34	Firenze	1,014,423	353.5692	89	Siracusa	402,822	156.7500
35	Foggia	628,556	1094.5580	90	Sondrio	181,437	109.1102
36	Forlì-Cesena	394,067	70.8847	91	Taranto	583,479	315.5909
37	Frosinone	493,067	85.0009	92	Teramo	309,859	77.3974
38	Genova	850,071	290.8360	93	Terni	228,218	56.8256
39	Gorizia	139,673	745.3390	94	Torino	2,277,857	149.2379
40	Grosseto	223,045	241.4419	95	Trapani	434,476	142.0155
41	Imperia	215,130	86.5403	96	Trento	538,604	148.9746
42	Isernia	85,805	56.41261	97	Treviso	885,972	171.6208
43	La Spezia	220,698	151.7241	98	Trieste	234,682	106.4168
44	L’Aquila	301,910	152.9389	99	Udine	531,466	98.4066
45	Latina	574,891	136.9423	100	Varese	890,043	66.7326
46	Lecce	802,082	341.3057	101	Venezia	854,275	40.4908
47	Lecco	339,238	2591.2880	102	Verbano-Cusio-Ossola	159,664	54.0219
48	Livorno	337,334	220.5977	103	Vercelli	173,868	76.5398
49	Lodi	229,338	206.9233	104	Verona	921,557	120.4661
50	Lucca	390,042	85.04473	105	Vibo Valentia	161,619	107.3836
51	Macerata	318,921	466.7020	106	Vicenza	865,082	103.4919
52	Mantova	412,610	248.8562	107	Viterbo	319,008	342.4964
53	Massa-Carrara	196,580	181.7439				
54	Matera	199,685	106.3000				
55	Medio Campidano	98,623	276.9579				

Three epidemiologic parameters are considered in this study: the *infection rate*, or *incidence of the pathology*, quantifying the pathology appearance frequency in a particular population (Shields and Twycross 2003), which is defined as the number of infected people in a province normalized to the province population; the *mortality rate* (Gülmezoglu et al. 2004), quantifying the frequency of occurrence of death in a defined population, which is defined as the number of deaths in a province normalized to the province population; and the *case fatality rate* (Harrington 2020), quantifying the frequency of occurrence of deaths from a specified pathology compared to the total number of people diagnosed with the pathology, which is defined as the number of reported deaths in a province normalized to the number of reported *cases.*

A statistical analysis is carried out to correlate the infection rate, the mortality rate, and the case fatality rate with PM_10_ concentrations. In the paper by Borro et al. (2020), PM_2.5_ pollution levels from a single station within each province territory were considered. The present study considers PM_10_ concentration measurements from all ground stations available within each Province territory, which allows accounting for the natural variability of the particulate matter pollution within the single province territories, including urban, semi-urban, and rural areas. In fact, particulate concentration variability within single province territories is an important aspect to be properly accounted for when correlating epidemiologic parameters with atmospheric pollution. For this purpose, particulate concentration variability within each single province territory is used as a weighting factor in the statistical analysis carried out to correlate epidemiologic parameters with PM_10_ concentrations. Specifically, we computed the average PM_10_ concentration value over the period 15–26 February for each station within each province territory. The mean and standard deviation of these time-averaged PM concentration values from the different stations within each province territory are used in the statistical analysis for comparison with the epidemiologic parameters.

A linear fit was applied to the mean PM_10_ and PM_2.5_ concentration values for the 110 Italian provinces over the period 15–26 February 2020, using a linear regression function with the form *Y* = *A* + *B* × *X*, with *A* being the mean PM_2.5_ concentration values and *Y* the corresponding mean PM_10_ concentration values, with the weight in the fit given by the error bars Δ*Y*. The regression analysis yielded the following results: *A* = 8.1 ± 0.5 μg/m^3^, *B* = 1.04 ± 0.02, correlation coefficient = 0.96 and *p* value < 0.0001. These results reveal a very high correlation between mean PM_10_ and PM_2.5_ concentration values in most provinces. This result testifies the simultaneous and co-located presence of both particle types, as expected, since PM_2.5_ particles are, by definition, included within PM_10_ particles and therefore PM_10_ concentrations are dependent on PM_2.5_ concentrations. Specifically, based on the above reported numbers, mean PM_10_ concentration values are found on average to be higher than corresponding PM_2.5_ values by ~ 8 μg/m^3^, with the concentration growth rates being almost identical for PM_2.5_ and PM_10_ particles (4% higher for PM_10_ particles with respect to PM_2.5_).

**Fig. 9:**
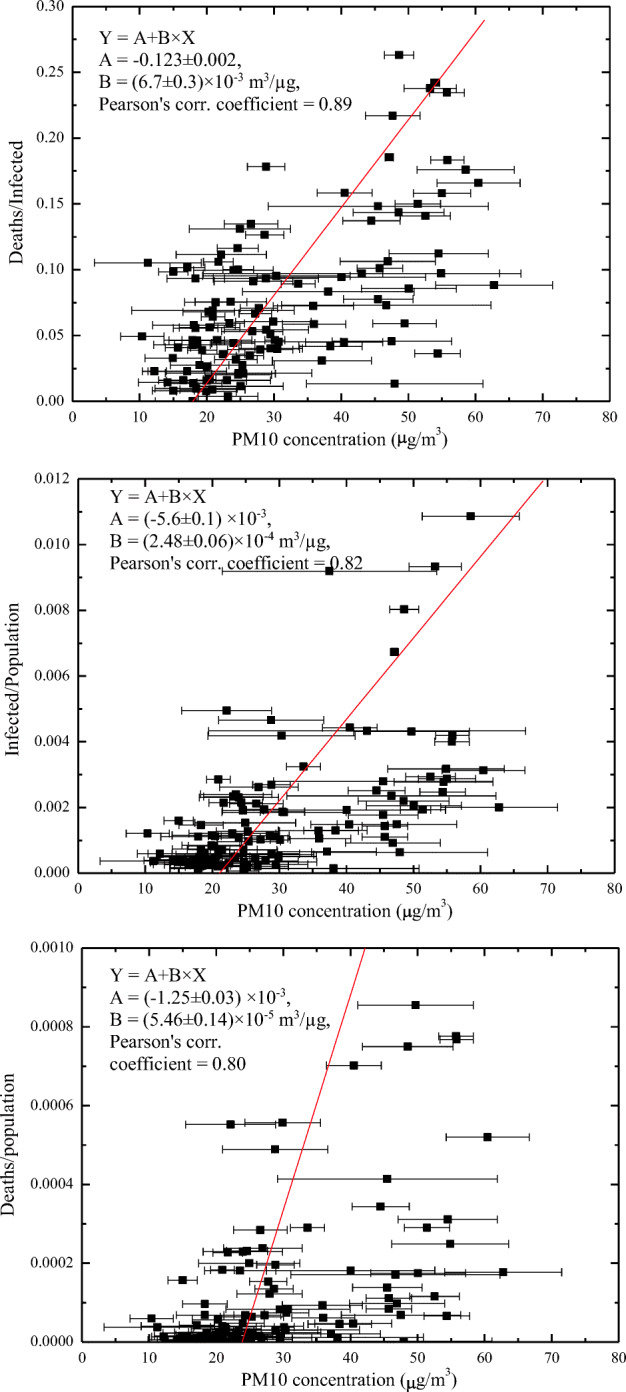
Linear regression analysis correlating the case fatality rate (upper panel), the incidence of the pathology (middle panel), and the mortality rate (lower panel) in the period 20 February–31 March 2020 with the average PM_10_ concentration values in the period 15–26 February 2020. The statistical analysis is extended over all 110 Italian provinces. Error bars represent the standard deviations of the PM concentration values in the different stations within each province territory

Coming to the results from the statistical analysis correlating epidemiologic parameters with PM_10_ concentrations, the upper panel of Fig. 9 compares the “case fatality rate” in the period 20 February–31 March 2020 with the corresponding average PM_10_ concentration values in the period 15–26 February 2020 for all 110 Italian provinces. A linear fit was applied to the data, using a linear regression function with the form *Y* = *A* + *B* × *X*, with *X* being the average PM_10_ concentration values and *Y* the corresponding “case fatality rate” values. Values of PM_10_ concentration variability within each province territory, i.e., its standard deviation, are included as error bars Δ*X* and are used as a weighting factor in the statistical analysis. The following results are obtained: *A* = (− 0.123 ± 0.002), *B* = (6.7 ± 0.3) × 10^−3^ m^3^/μg, with the Pearson’s correlation coefficient being equal to 0.89 and the Spearman’s correlation coefficient being equal to 0.87. The *p* value is smaller than 0.0001, which indicates less than 0.01% probability that no statistically significant relationship is present between the two compared quantities. A slope of the regression line of the “case fatality rate” vs. PM_10_ concentration of 6.7 × 10^−3^ μg/m^3^ implies a doubling (from 3 to 6%) of the mortality rate of infected patients for an average PM_10_ concentration increase from 22 to 27 μg/m^3^.

The middle panel of Fig. 9 compares the “incidence of the pathology” in the period 20 February–31 March 2020 with the corresponding average PM_10_ concentration values in the period 15–26 February 2020 for the 110 Italian provinces. The best-fit statistical analysis carried out to correlate these two parameters, with *X* being the average PM_10_ concentration values and *Y* the corresponding “incidence of the pathology” values, indicates a regression line with *A* = (-5.6 ± 0.1) × 10^−3^ and *B* = (2.48 ± 0.06) × 10^−4^ m^3^/μg, a Pearson’s correlation coefficient equal to 0.82, a Spearman’s correlation coefficient equal to 0.79, and *p* value < 0.0001. A slope of the regression line of the “incidence of the pathology” vs. PM_10_ concentration of 2.48 × 10^−4^ m^3^/μg implies a doubling (from 1 to 2‰) of the incidence of the pathology for an average PM_10_ concentration increase from 25 to 29 μg/m^3^.

The lower panel of Fig. 9 compares the “mortality rate” in the period 20 February–31 March 2020 with the corresponding average PM_10_ concentration values in the period 15–26 February 2020 for the 110 Italian provinces. The linear regression analysis, with *X* being the average PM_10_ concentration values and *Y* the corresponding “mortality rate” values, leads the following results: *A* = (− 1.25 ± 0.03) × 10^−3^ and *B* = (5.46 ± 0.14) × 10^−5^ m^3^/μg, a Pearson’s correlation coefficient equal to 0.80, a Spearman’s correlation coefficient equal to 0.79, and *p* value < 0.0001. The slope of the regression line of the “mortality rate” vs. PM_10_ concentration is (5.46 ± 0.14) × 10^−5^ m^3^/μg, which implies a tripling (from 0.1 to 0.3‰) of the mortality rate for an average PM_10_ concentration increase from 25 to 29 μg/m^3^.

The above results reveal the presence of much higher correlation coefficients than those reported in the paper by Borro et al. (2020) (0.89 against 0.7 for the “case fatality rate” vs. PM concentrations, 0.80 against 0.65 for the mortality rate vs. PM concentrations, and 0.82 against 0.67 for the incidence of the pathology vs. PM concentrations). The higher values found in the present correlation analyses are to be attributed to several motivations. First, the present correlation analyses are considering PM_10_ particles instead of PM_2.5_ particles, the formers acting as a more efficient virus carriers (see section 3.1). Secondly, the present analyses are considering PM concentration values, which properly account for their variability within the single province territories, using this variability as a weighting factor in the regression analysis. This implies that data points characterized by a higher variability of PM concentrations are considered with a lower weight in the best-fit analysis. This approach is quite effective in properly filtering potential biases or outliers associated with the use of a single pollution monitoring station in each province territory, especially in those cases when PM pollution levels sensitively vary within the province territory. Correlation coefficient values in the range 0.80–0.89 testify a high statistical significance. The correlation coefficient quantifies the strength and direction of the linear relationship between two variable quantities and the reliability of the linear model depends on the number observed data points. Thus, both the correlation coefficient value and the number of considered data points need to be properly accounted for in the assessment of the significance of the results. In general, the larger is the number data points, the lower is the acceptable correlation coefficient.

Based on the above analyses’ results, it appears clear that the most effective epidemiological parameter to assess the impact of particulate pollution on COVID-19 pandemic is the case fatality rate. In fact, results from the analysis reveal that case fatality rate is the epidemiologic parameter characterized by the highest correlation with PM_10_ concentrations, and consequently is the epidemiologic parameter most affected by particulate pollution. This is also coherent with the possible interpretation of the results in terms of biological response of the human respiratory system to particulate pollution exposition. In this regard it should be noted that, during the second pandemic wave in Autumn 2020, the number of infected people has strongly increased over the entire Italian territory, also as a result of the increased number and higher distribution of virus hotbeds, with the geographical distribution of infected patients becoming almost evenly distributed over the Italian territory. However, what has remained substantially unchanged with respect to the first pandemic wave is the case fatality rate, with strongly unbalanced values between high and low polluted areas.

Correlations between the different epidemiologic parameters and PM concentrations are strongly dependent on the elapsed time lag considered between the pollution events and the time window considered for epidemiologic parameters, as well as on the duration of the time window considered for the pollution event. In general, the consideration of the presence of a possible time gap between population exposure to enhanced PM concentrations and the onset of the infection, and the eventual death of patients, ensures that the pollution exposition period is long enough to induce a biological response in human tissues. A sensitivity study was carried out considering different time gaps and pollution integration times. Unfortunately, in the sensitivity analysis, there was no possibility to also vary the time window considered for the assessment of the epidemiologic parameters, as in fact values for these parameters were provided by the Italian Statistics Institute only for the period 20 February–31 March 2020 (ISTAT 2020). Specifically, we considered three different time windows for particulate pollution: 01–26 February 2020, 01–19 February 2020, and 15–26 February 2020. The first time window allows including all three major pollution outbreaks in February 2020 identified in Figs. 6 and 7, i.e., the periods 06–11 February, 15–19 February, and 20–26 February. The second time window includes only the first two pollution outbreak events, while the third time window includes the last two pollution outbreak events. Results reported in Table 2, which focus specifically on the "case fatality rate", reveal a maximum correlation when comparing the epidemiologic parameters in the period 20 February–31 March 2020 vs. the corresponding average PM concentration values observed in the period 15–26 February 2020. The table also lists the values of the time intervals elapsed from infection to death, which are computed from the central day of the considered pollution time window and the central day of the time interval considered for the epidemiologic parameter (20 February–31 March 2020). Maximum correlation is observed for an elapsed time of 20 days.

**Table 2:** Results for the regression analysis, expressed in terms of Pearson’s correlation coefficient, considering different elapsed time lags between the pollution events and the time interval considered for the “case fatality rate.” The table also lists the times elapsed from infection to death, which are computed from the central day of the considered pollution time window and the central day of the time interval considered for the “case fatality rate” (20 February–31 March 2020)

	Particulate pollution time window	Pearson’s correlation coefficient	Time elapsed since infection (days)
1	01–26 February 2020	0.72	27
2	01–19 February 2020	0.70	31
3	15–26 February 2020	0.89	20

The revealed positive correlation between epidemiologic parameters and PM_10_ concentrations identified in the present research effort does not imply a direct and univocal cause-effect relation, but it indicates that PM pollution is certainly one of the several factors that influenced the pandemic outbreak in Northern Italy in the period February–March 2020. In principle, some other circumstance (for example, the effect of meteorological variables) could have caused both epidemiologic factors and PM_10_ concentrations to change. With regard to this aspect, the reader should be aware that the reported correlation is to be interpreted in a mathematical sense, i.e., it testifies a co-occurrence of low/high values of COVID-19 epidemiologic parameters and low/high pollution levels.

The possible occurrence of spurious correlations has to be carefully accounted for (Bolton 1994). In statistics, a spurious correlation refers to a connection between two variables which may be caused by a third factor that may be not apparent at the time of analysis, sometimes called a confounding factor. There are numerous approaches to spot spurious correlations. Among others: ensuring a proper representative sample, obtaining an adequate sample size, being wary of arbitrary endpoints, using a null hypothesis and checking for a low *p* value. In the present statistical analysis, the availability of a representative data sample is ensured, as in fact the considered epidemiologic parameters’ data and PM measurements are considered for the entire Italian territory and population and not only for a subset of these. For most statistical applications, the sample size is considered adequate when exceeding 100 elements (Francis et al. 2010; Sandelowski, 1995; Malterud et al. 2016). The endpoints in our study are the considered epidemiologic parameters, i.e., the case fatality rate and the infection and mortality rates, which are well-established clinical events or outcomes that can be objectively measured (Pilz et al. 2012). *p* values smaller than 0.0001 where found in all statistical analyses correlating epidemiologic parameters with PM_10_ concentrations. The above arguments testify that in our analysis, the occurrence of spurious correlation is very unlikely.

**Fig. 10:**
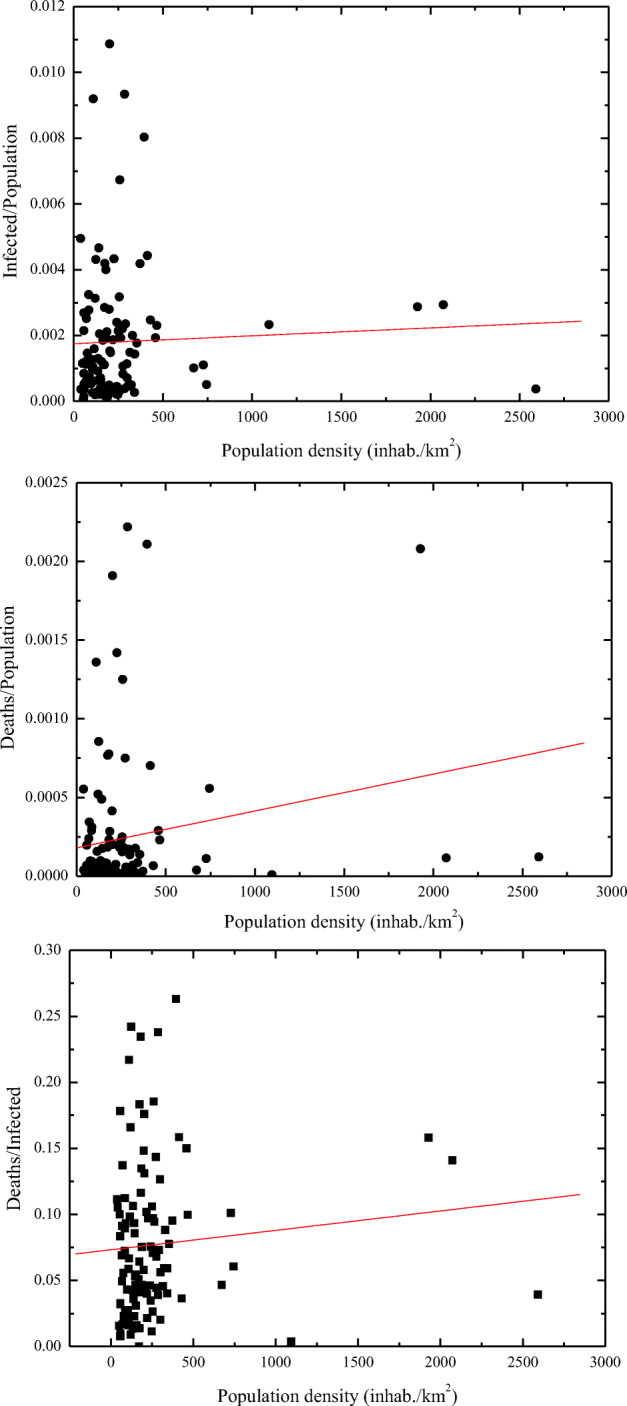
Linear regression analysis correlating the case fatality rate (upper panel), the incidence of the pathology (middle panel), and the mortality rate (lower panel) in the period 20 February–31 March 2020 with the population density. The statistical analysis is extended over all 110 Italian provinces

The effect of population density on epidemiologic parameters has also been investigated. Results indicate that this effect is quantitatively far less important than PM pollution. Specifically, Fig. 10 shows the linear regression analysis correlating population density with the incidence of the pathology (upper panel), the mortality rate (middle panel), and the case fatality rate (lower panel) in the period 20 February–31 March 2020. This analysis is again extended over all 110 Italian provinces. Specifically, the statistical analysis correlating the incidence of the pathology with population density reveals a totally missing correlation, with a Pearson’s correlation coefficient of 0.045 and a Spearman’s correlation coefficient of 0.17 (Fig. 10, upper panel). The *p* value is equal to 0.65, which indicates 65% probability that no statistically significant relationship is present between the two compared quantities. The statistical analysis correlating the mortality rate with population density reveals a very low correlation, with a Pearson’s correlation coefficient of 0.19, a Spearman’s correlation coefficient of 0.24, and a *p* value equal to 0.051 (Fig. 10, middle panel). Finally, the analysis correlating the case fatality rate with population density reveals an almost totally missing correlation, with a Pearson’s correlation coefficient of 0.093, a Spearman’s correlation coefficient of 0.18, and a *p* value equal to 0.34 (Fig. 10, lower panel). The above results indicate that correlations between epidemiologic parameters and population density are not significant.

The reported statistical results do not completely exclude a correlation between the abovementioned epidemiologic parameters and population density: they only underline that there are so many outliers in the analysis to make this correlation meaningless. More specifically, the different panels of Fig. 10 reveal the presence of data points with a highly scattered distribution. This implies that outliers are present in the analysis, i.e., there is a number of provinces with low population density and high values of the epidemiologic parameters and a number of provinces with high population density and low values of the epidemiologic parameters, and the presence of these data points is severely compromising the regression analysis. For example, provinces such as Napoli, Monza, and Trieste are characterized by quite high population densities, but have low values for the epidemiologic parameters and were only slightly affected by COVID-19. Analogously, provinces such as Cremona, Lodi, and Piacenza are characterized by low population densities, but were severely affected by COVID-19. Obviously, few outliers were also present in the statistical analysis correlating the epidemiologic parameters with PM_10_ concentrations (for example, the province of Aosta in Valle d’Aosta, where a large number of hospitalizations refer to patients coming from other areas of the Italian territory as a result of their short-term mobility associated with winter skiing holidays). However, these outliers were very few and their presence only slightly affected the results of the regression analysis.

## Summary and final remarks

The devastating impact in terms of number of infected people and deaths associated with the first wave of the COVID-19 pandemic in the early portion of 2020 was the result of a variety of contributing causes and circumstances. While the spread and effective impact of the SARS-CoV-2 virus in Northern Italy was primarily related to the lifestyles and social habits of the different communities and the presence of specific infection hotbeds triggered by individual infected patiens often returning from travels abroad, environmental and meteorological factors have possibly also played a role.

In the present paper, we illustrated the evolution of PM_2.5_ and PM_10_ concentrations in Northern Italy throughout the month of February 2020, identifying in the central part of the month the presence of enhanced PM_2.5_ and PM_10_ concentrations over large portions of the Po Valley, with levels exceeding 60 μg/m^3^ for both species. A marked reduction of pollution levels was observed in the final part of the month, few days after the shutdown of all vehicular and industrial activities associated with the lockdown in Northern Italy started on 25 February 2020, with PM_2.5_ and PM_10_ concentrations abruptly dropped to levels not exceeding 15–20 μg/m^3^ for both species.

Computations were carried out using an analytical microphysical model capable to simulate the different coagulation processes (Brownian diffusion, laminar shear, turbulent fluctuations, uniform horizontal motion, and gravitational and drag forces) occurring during the formation of virus-transmitting PM particles. The simulation model uses as input data PM_2.5_ and PM_10_ pollution measurements and specific literature information on exhaled particles’ sizes and concentrations, their residence time, transported viral dose, and minimum infective dose. Model results allow to affirm that airborne transmission through virus-laden PM particles, and its capability to convey SARS-CoV-2 into the human respiratory system, may have played an important role in the early 2020 pandemic outbreak of COVID-19 infection in Northern Italy. This statement is supported by the model results illustrated in Section 4, which clearly indicate that the number of small breathing and coughing particles coalescing on PM_2.5_ or PM_10_ particles may exceed the minimum infective dose of SARS-CoV-2 and other respiratory viruses.

While airborne transmission through virus-laden PM particles is a possible mechanism to convey viable amounts of SARS-CoV-2 into the human respiratory system, which is likely to happen in high PM pollution conditions, the more traditional airborne transmission directly through the inalation of infectious briefing and coughing particles exhaled by symptomatic and or asymptomatic patients, especially in case of prolonged permanence in indoor unventilated spaces, is a very likely process to take place (Morawska and Cao 2020), especially if the preliminary emerging research results on the high viral load and infertility of SARS-COV-2 will be confirmed. A deeper insight into these mechanisms should be the object of future dedicated research efforts.

In the paper, we have also reported the results from a statistical analysis correlating the infection rate, or incidence of the pathology, the mortality rate, and the case fatality rate with PM concentrations, which reveals a high correlation of these epidemiologic parameters with PM_10_ concentrations (correlation coefficients in the range 0.80–0.89), with the case fatality rate doubling (from 3 to 6%) for an average PM_10_ concentration increase from 22 to 27 μg/m^3^ and the infection rate doubling (from 1 to 2‰) and the mortality rate a tripling (from 0.1 to 0.3‰) for average PM_10_ concentration increase from 25 to 29 μg/m^3^. Epidemiologic parameters’ data were also compared with population density data, but no clear evidence of a mutual correlation between these quantities was found.

Correlations between epidemiologic factors and PM concentrations do not imply a direct and univocal cause-effect relationship between PM pollution and the onset of COVID-19 pandemic. The reported correlation has to be interpreted in a mathematical sense, i.e., it testifies the simultaneous co-occurrence of low/high values of both COVID-19 epidemiologic parameters and pollution levels. In the interpretation of the meaning of the high Pearson’s and Spearman’s correlation coefficient values obtained in the present study, the possible occurrence of spurious correlations due to indirect causes or remote mechanisms has to be carefully accounted for (Bolton 1994). Nevertheless, results from this paper clearly testify that PM pollution is one of the several factors, certainly not a secondary one, which affects COVID-19 incidence. In order for PM pollution to play a role, a number of virus hotbeds need to distributed over the territory. For example, PM_10_ concentrations in excess of 100 μg/m^3^ were reported in late 2019/early 2020 in several metropolitan cities in China (Beijing, Nanjing, Shanghai, Tianjin, Guangzhou). However, none of these metropolitan cities was affected by a COVID-19 pandemic outbreak and spread similar to the one which exploded in Wuhan (Hebei Region, China) in late 2019, possibly because of the missing presence and concurrence of specific virus hotbeds.

A more quantitative assessment of the contributing role of PM pollution on early 2020 COVID-19 outbreak in Northern Italy implies further dedicated studies, possibly using additional experimental data, with statistics representing one of the several needed tools to be used in the investigation. The experimental/modelling evidence reported in this paper certainly calls for additional research aimed at quantitativly assessing all possible contributing causes based on dedicated sensitivity analyses. Particularly interesting in this regard is the dataset presently under collection during the second pandemic wave in Italy and future research work will focus on identifying possible correlations between the epidemiologic parameters and particulate matter pollution levels for this forthcoming dataset.
